# Immunocompetent mouse allograft models for development of therapies to target breast cancer metastasis

**DOI:** 10.18632/oncotarget.15695

**Published:** 2017-02-25

**Authors:** Yuan Yang, Howard H. Yang, Ying Hu, Peter H. Watson, Huaitian Liu, Thomas R. Geiger, Miriam R. Anver, Diana C. Haines, Philip Martin, Jeffrey E. Green, Maxwell P. Lee, Kent W. Hunter, Lalage M. Wakefield

**Affiliations:** ^1^ Lab of Cancer Biology and Genetics, Center for Cancer Research, National Cancer Institute, Bethesda, MD, USA; ^2^ High Dimension Data Analysis Group, Center for Cancer Research, National Cancer Institute, Bethesda, MD, USA; ^3^ British Columbia Cancer Agency, Vancouver Island Center, Victoria, British Columbia, Canada; ^4^ Pathology Histotechnology Lab, Leidos Biomedical Research Inc., Frederick National Laboratory for Cancer Research, Frederick MD, USA

**Keywords:** metastasis, breast cancer, immunocompetent mouse models, genomics, experimental therapeutics

## Abstract

Effective drug development to combat metastatic disease in breast cancer would be aided by the availability of well-characterized preclinical animal models that (a) metastasize with high efficiency, (b) metastasize in a reasonable time-frame, (c) have an intact immune system, and (d) capture some of the heterogeneity of the human disease. To address these issues, we have assembled a panel of twelve mouse mammary cancer cell lines that can metastasize efficiently on implantation into syngeneic immunocompetent hosts. Genomic characterization shows that more than half of the 30 most commonly mutated genes in human breast cancer are represented within the panel. Transcriptomically, most of the models fall into the luminal A or B intrinsic molecular subtypes, despite the predominance of an aggressive, poorly-differentiated or spindled histopathology in all models. Patterns of immune cell infiltration, proliferation rates, apoptosis and angiogenesis differed significantly among models. Inherent within-model variability of the metastatic phenotype mandates large cohort sizes for intervention studies but may also capture some relevant non-genetic sources of variability. The varied molecular and phenotypic characteristics of this expanded panel of models should aid in model selection for development of antimetastatic therapies *in vivo*, and serve as a useful platform for predictive biomarker identification.

## INTRODUCTION

Metastasis is the most lethal aspect of the carcinogenic process, and the prognosis for patients with disseminated disease at diagnosis is dismal, with five-year survival rates of less than 25% in breast cancer [1]. While significant progress has been made in treatment of localized disease [1], results of three decades worth of randomized clinical trials in breast cancer patients receiving adjuvant chemotherapy who went on to develop metastases showed no evidence for an impact of subsequent therapy on patient survival [2]. Thus there is clearly a major problem with our current approaches to the development of therapeutics that effectively target the metastatic process.

The preclinical process for target identification and drug development in cancer has been justly criticized for the poor translatability of results into clinically useful practice [3]. There are many factors that play into this problem, but one limitation of most preclinical therapeutic studies is that they have historically focused on effects of the intervention on the primary tumor. Metastasis burden, or survival endpoints driven by metastatic disease, are rarely used in preclinical drug testing. Furthermore, the information that we have about the biology of the metastatic process and the response of metastases to therapy is generalized from a very small number of widely-used models. In breast cancer, these include the MDA-MB-231 human breast cancer cell line xenografted into immunodeficient mice (see eg. [4, 5]), the 4T1 murine mammary cancer cell line allografted into syngeneic immunocompetent mouse hosts [6], and the genetically engineered MMTV-PyVT mouse model of metastatic breast cancer [7]. While these models have indisputably generated many useful mechanistic insights, they do not begin to capture the heterogeneity of human breast cancer.

Metastasis is a highly complex multi-step process that involves a continual and reciprocal dialog between the tumor cells and the systemic and local microenvironments [8]. The steps involved include escape of tumor cells from the primary tumor site by intravasation, passage through the circulation to distant organs, extravasation and successful colonization of the distant site. Every step poses multiple challenges to the survival of the tumor cell, which has to face and evade threats that include loss of attachment-based survival signals, physical damage, active immune surveillance and consequences of interacting with inhospitable microenvironments to which it is not adapted. Thus in an ideal world, therapeutic intervention studies for metastasis would be performed in autochthonous models, such as the genetically engineered mouse (GEM) models, where the tumor and host components can co-evolve as they would for the human disease. However, there are very few existing GEM models of breast cancer that metastasize with a reasonable efficiency. The most widely-used metastatic GEM model of breast cancer is the MMTV-PyVT model, in which female mice show an ~90% incidence of lung metastases by 100 days of age [7]. The MMTV-Neu model is also metastatic, but with a lower incidence (~50%) and with a longer time-frame to development of clinically significant metastatic disease ( > 1 year) [9]. Metastasis from other intact GEM models is even less efficient, making GEM models more useful for natural history studies of the metastatic process than for therapeutic intervention studies.

For the purposes of cost-effective drug screening *in vivo*, metastatic models with a high efficiency of metastasis and a shorter time-frame are needed. Currently these requirements can only be met using transplantation models. Ideally such models should capture at least some of the heterogeneity of human breast cancer. Additionally, it is increasingly appreciated that the full efficacy of conventional and targeted therapeutic approaches frequently depends in part on activation of anti-tumor immune responses [10]. Thus models with an intact immune system are desirable for development of most therapeutic strategies, not just immunotherapy. To address some of these issues in breast cancer, we have assembled a panel of 12 metastatic mouse mammary tumor cell line models for use in syngeneic, fully immunocompetent hosts. Here we describe the clinico-pathologic, genomic and transcriptomic characterization of these models. We explore their relationship to human breast cancer and address their advantages and challenges for the development of anti-metastasis therapies.

## RESULTS

### Origins and metastatic properties of the model panel

To assemble the panel, we searched the literature for reports of mouse mammary tumor cell lines with demonstrated metastatic ability, and we then obtained the lines directly from the originating laboratory/investigator so as to avoid problems with inter-laboratory subline drift. The origins of the 12 cell lines that comprised the metastatic mouse mammary tumor model panel are summarized in Table 1. Half the models were derived from spontaneously arising mammary tumors, and half were derived from genetically engineered mouse models (GEMM). Four different mouse strain backgrounds are represented in the panel. In these studies, we focused on lung as the predominant metastatic site, since metastases to other sites were only rarely seen following orthotopic implantation. The conditions in our laboratory that were used to generate metastases from each model, and the efficiency of lung metastasis in that format are summarized in Supplementary Table 1. Where possible, models were used in an orthotopic implantation format with surgical resection of the primary tumor at 7-10mm in diameter. However, we were unable to get all models to metastasize efficiently with this approach, in which case primary tumors were either left unresected, or the tumor cells were delivered by tail-vein injection, as indicated in the table. Some models that were described in the original publication as metastasizing from the orthotopic site did not do so efficiently in our hands (Eg. TSAE1). Contributing factors are likely to include mouse substrain drift leading to minor histocompatibility antigen mismatches, and inter-institutional variation in factors such as mouse diet, housing conditions, and microbiota. In general we find the metastatic phenotype to be much more sensitive to environmental and immunologic factors than the primary tumor phenotype, and our experience is that the same model can exhibit very different metastatic efficiencies in different facilities, despite using host mice from the same supplier and cells prepared under identical conditions. Thus each model should be further optimized for the facility in which it will be used. Models that require tail-vein injection can be adapted to orthotopic implantation by repeated rounds of selection *in vivo*, but the results presented here represent the unmodified models from the original sources.

**Table 1:** Origin of cell lines used in the metastatic mammary tumor panel

Cell line designation in original publication	Simplified designation	Mouse strain	Tumor origin of cell line	Driver oncogenic event	Brief description of cell line origin	Ref.	Investigator source of cell lines used in analysis
4T1	4T1	BALB/c	Spont	Unknown	Derived from spontaneous tumor arising in a BALB/cfC3H mouse; selected as spontaneously resistant to thioguanine	[6]	Dr. Fred Miller, Karmanos Cancer Institute, Detroit
6DT1	6DT1	FVB/N	GEMM	Myc overexpression	Derived from mammary tumor arising in MMTV-Myc transgenic mouse	[55]	Dr. Robert Dickson**, Georgetown University Medical Center, Washington DC, USA
D2A1	D2A1	BALB/c	Spont	Unknown	Derived from spontaneous mammary tumor originating from a D2 hyperplastic alveolar nodule line	[56]; [57]	Dr. Ann Chambers, London Regional Cancer Center, London, Ontario, CANADA
E0771	E0771	C57BL/6	Spont	Unknown	Derived from a spontaneous adenocarcinoma in the mammary gland of a C57Bl/6 mouse.	[16]; [12]	Drs. Fengzhi Li/Enrico Mihich, Roswell Park Cancer Institute, Buffalo, NY, USA
EMT6	EMT6	BALB/c	Spont	Unknown	Derived from primary mammary tumor KHJJ arising in BALB/c mouse after implantation of a hyperplastic alveolar nodule. EMT6 was selected in culture from the 25th transplant generation of KHJJ	[58]	Dr. Sara Rockwell, Yale Univ, New Haven, USA
F311	F311	BALB/c	Spont	Unknown	Sarcomatoid clone derived from a transplantable ER-negative mammary adenocarcinoma (M3) that arose spontaneously in a Balb/c mouse.	[59]	Dr. Daniel Alonso, Quilmes National University, Buenos Aires, ARGENTINA
HRM-1	HRM1	FVB/N	GEMM	Mutant PIK3CA	Derived from a recurrent tumor in a PIK3CA-H1047R inducible transgenic mouse in which the tumor partially regressed and then recurred following transgene shutoff by Dox withdrawal.	[60]*	Dr. Jean Zhao, Dana-Farber Cancer Institute, Boston, USA
M6	M6	FVB/N	GEMM	Functional inactivation of p53 and Rb	Derived from mammary tumor arising in a C3(1)TAg transgenic mouse	[61]	Dr. Jeffrey Green, National Cancer Institute, Bethesda, USA
Met-1	MET1	FVB/N	GEMM	Functional activation of PI3K pathway	Derived from a transplanted MMTV-PyVT mammary tumor passaged in mammary fat pad	[62]	Dr. Alexander Borowsky, UC Davis, Sacramento, USA
MVT1	MVT1	FVB/N	GEMM	Myc and VEGFA overexpression	Derived from mammary tumor arising in MMTV-Myc-VEGF bitransgenic mouse	[55]	Dr. Robert Dickson**, Georgetown University Medical Center, Washington DC, USA
r3T	R3T	129S3	GEMM	Unknown but with contributions from Src and Ras pathway activation	Parental cell line was derived from a mammary tumor in OPN knockout mice induced by MPA pellets followed by DMBA administration. The line was transformed with PyMT and activated Ras, and a derivative of the transfomed line was re-isolated from fat pad tumors (r3T).	[17]	Dr. Susan Rittling, Forsyth Institute, Cambridge, USA
TS/A-E1	TSAE1	BALB/c	Spont	Unknown	Parental TS/A cell line was derived from a spontaneous mammary tumor arising in a retired BALB/c breeder mouse. TS/A-E1 is a subclone of TS/A line with epithelial morphology and higher metastatic potential.	[63]	Dr. Carla di Giovanni, Univ of Bologna, Bologna, ITALY

Notes: Spont = spontaneous tumor; GEMM=genetically engineered mouse model; *Description of GEMM model as cell line is unpublished; **Deceased

**Figure 1:**
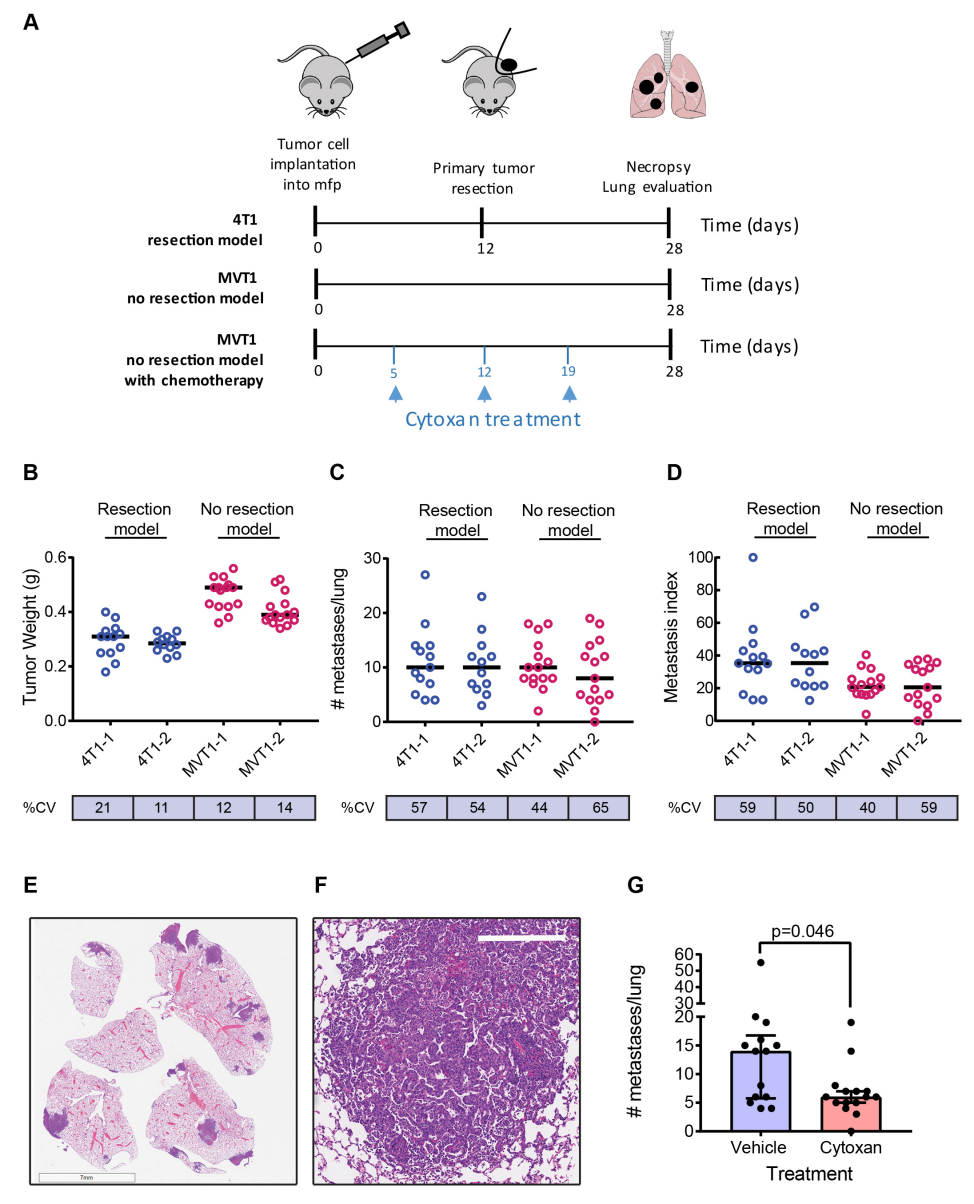
Metastasis in representative models **A**. Timelines for a representative resection model (4T1), and a no resection model (MVT1) with or without cytoxan treatment. **B**.-**D**. Representative results from two independent experiments for each model. B, Tumor weights at resection (4T1) or endpoint (MVT1); C, number of metastases/lung; D, metastatic index, calculated as number of metastases normalized to weight of primary tumor. Black bars show the median values. % CV is the within-cohort % coefficient of variation. **E**. Low power image of metastases in lung lobe sections for the MVT1 model (scale bar is 7mm). **F**. High power image of MVT1 lung metastasis (scale bar is 300μm). **G**. Effect of cytoxan treatment on metastatic burden in the MVT1 model. Results are median +/− interquartile range (*n* = 15/group); Mann-Whitney test.

Metastatic characteristics of two representative models are shown in Figure 1. The 4T1 model is a resection model in which the primary tumor is resected when it reaches 7-10mm diameter, while the MVT1 model is a no-resection model with the primary tumor left in until the study endpoint in order to get a high metastatic burden (Figure 1A). In general, the primary tumor weights at resection (4T1) or at endpoint (MVT1) show relatively low variation within a cohort, with a % coefficient of variation (% CV) ranging from ~10-20% (Figure 1B). Results of two independent experiments are shown for each model. In contrast, the metastatic burden is an intrinsically very variable phenotype even under highly controlled conditions, with % CV ranging from ~40-65% (Figure 1C). Similar variability is seen in both resection and non-resection models suggesting that the resection surgery itself is not introducing additional variability. Furthermore, the variability is not decreased by normalizing the metastasis burden to the size of the matched primary tumor (Figure 1D), suggesting that variations in primary tumor size do not contribute in a major way to the within-cohort variation in metastatic burden. Representative low power (Figure 1E) and high power (Figure 1F) images of lung metastases from the MVT1 model are shown. The high intrinsic variability of the metastatic phenotype has practical consequences for the design of intervention experiments. A power calculation using the variance that we see in the 4T1 and MVT1 models shows that an absolute minimum of 12 mice/group must be used for a two group comparison in order to detect a 2-fold reduction of metastatic burden with a *p*-value < 0.05 and power of 80%. We typically use 15 mice/group. An example is shown for the effect of cytoxan treatment on metastatic burden in the MVT1 model (Figure 1G). Thus study cohorts for metastasis intervention studies must be considerably larger than is commonly used when primary tumor volume is the endpoint.

### Histopathology of primary tumors and lung metastases

**Figure 2:**
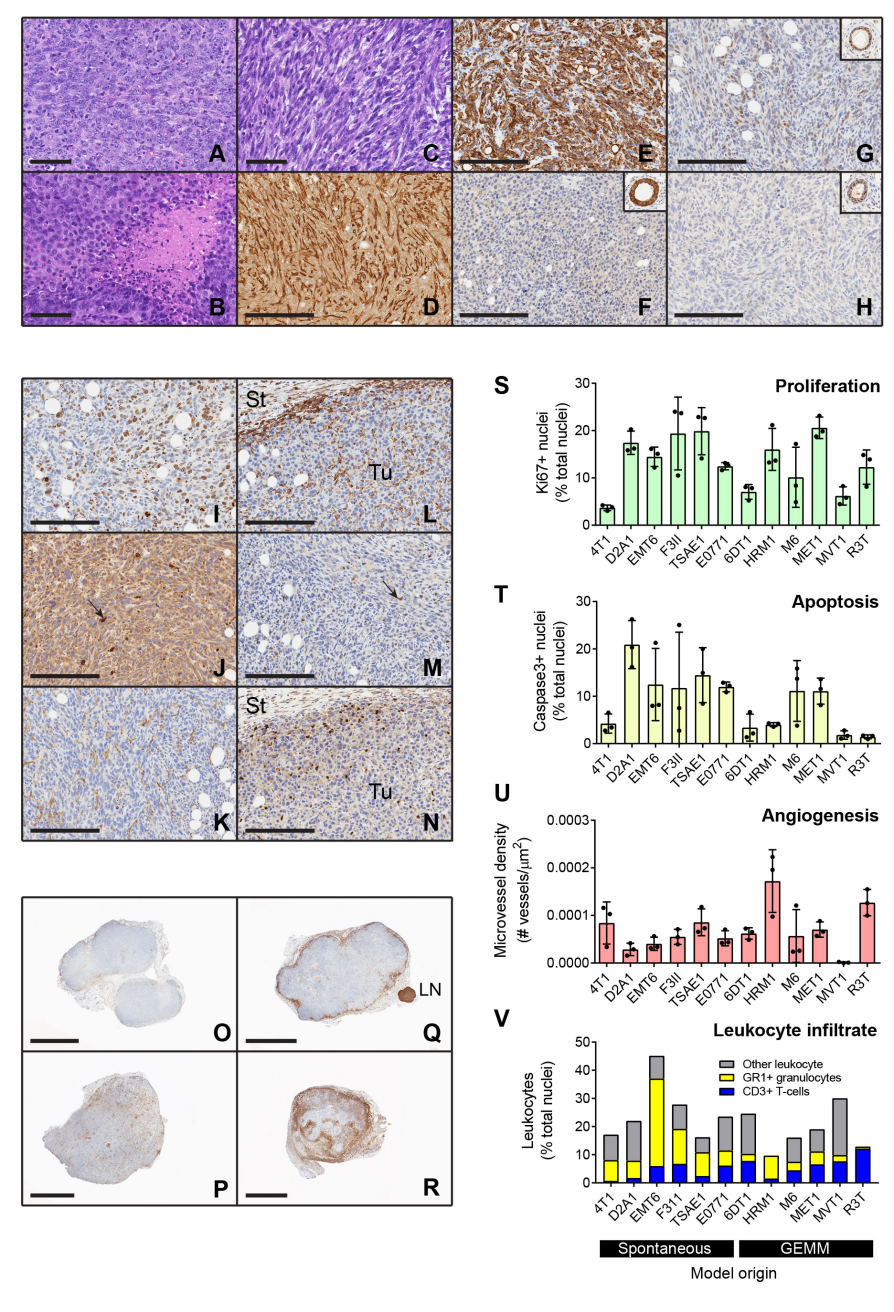
Histologic and immunohistochemical characterization of transplantable mouse mammary tumors **A**.-**C**. Histology of representative tumors including: A, 6DT1 poorly differentiated carcinoma; B, MET1 poorly differentiated carcinoma with areas of focal necrosis; and C, D2A1 spindle cell carcinoma. **D**.-**H**. Immunostaining for epithelial and mesenchymal markers and hormone receptors. Spindle cell tumors such as F3II (D) have cells that are positive for α-smooth muscle actin. However, nearly all tumors including those with a spindled morphology were positive for cytokeratin 8, shown for F3II (E). The one exception was E0771 (F) which was cytokeratin 8 negative but did not have a spindled histology. Inset shows normal mammary gland positive control from same slide. G-H. Hormone receptor staining. Four of the models were weakly positive for estrogen receptor, shown here for TSAE1 (G), while none were positive for progesterone receptor, as shown for TSAE1 (H). Insets show positive normal mammary glands from same slides. Scale bars are 60μm for A-C and 200μm for D-H. **I**.-**N**. Representative staining patterns for immunohistochemical markers of biological properties and immune infiltration in a TSAE1 primary tumor. (I), Nuclear Ki67 proliferation marker; (J), activated caspase-3 apoptosis marker; (K), CD34 angiogenesis marker; (L), CD45 pan-leukocyte marker; (M), CD3 T cell marker; (N), Ly6G granulocyte marker. Tu, tumor; St, stroma. Scale bars represent 200μm. Arrows show positive cells. **O**.-**R**. Low power views showing different patterns of CD45+ leukocyte infiltration into the tumors. O, HRM1 showing little peripheral accumulation or infiltration of leukocytes; P, MVT1 tumor showing infiltration with little peripheral accumulation; Q, MET1 tumor showing strong peripheral accumulation; R, EMT6 tumor showing strong peripheral accumulation and infiltration. LN, lymph node. Scale bars are 3mm for HRM1, MVT1, MET1 and 2mm for EMT6. **S**.-**V**. Quantitation of immunostaining data for three representative tumors/model. Bars show mean +/− SD. Models are ordered by the nature of their originating tumor type (spontaneous *vs* genetically-engineered mouse model, GEMM). In the leukocyte infiltration stackplot, mean values/model are plotted and the category of “other leukocytes” represents CD45+ cells that are not either T-cells (CD3+) or granulocytes (Ly6G+).

The histology of the primary tumors generated by the cell line models was assessed by a panel of veterinary pathologists and a human anatomic breast cancer pathologist to arrive at a consensus diagnosis. Tumors were described as carcinomas with either a spindle cell/sarcomatoid or a poorly differentiated histopathology (Figure 2A-2C; Supplementary Figure 1 and Supplementary Table 2). Despite the poorly differentiated and/or spindled morphology and the staining of some tumor cells for α-smooth muscle actin (Figure 2D), tumor cells from all but one of the models were immunohistochemically positive for cytokeratin 8 (CK8), confirming that they are epithelial in origin and not mouse fibroblast-derived (Figure 2E and Supplementary Figure 2). The E0771 model was essentially negative for CK8, but the cuboidal morphology of the tumor cells suggests they are not fibroblast-derived (Figure 2F, Supplementary Figure 1). Many of the primary tumors showed extensive areas of necrosis, including most strikingly the E0771 model which was highly necrotic even at small tumor size.

### Immunohistologic characterization of primary tumors

The hormone receptor status of the models was determined by immunostaining the primary tumors for estrogen receptor (ER) and progesterone receptor (PR). Cytokeratin 8 staining on adjacent sections allowed tumor cells to be distinguished from mouse stromal cells, since mouse stromal cells show some ER positivity [11]. Four tumor models were found to be weakly ER positive (EMT6, F3II, TSAE1 and HRM1), with Allred scores of 3-4 (shown for TSAE1 model in Figure 2G). None of the models was positive for PR (Figure 2H). The E0771 model has been referred to as ER+ [12], but it was derived and characterized in the 1950s before reliable ER assays were available, and it did not show ER positivity in our hands or those of our collaborators. Of the four ER+ tumor models, only TSAE1 was clearly growth-stimulated by estradiol *in vitro* (Supplementary Figure 3).

To address other biological properties of the models, immunohistochemical assessment of proliferation, apoptosis, angiogenesis and immune cell infiltration was also performed on the primary tumors (Figure 2I-2R). Tumor cell proliferation varied over a ~ 5x range between models (Figure 2S; models grouped by origin from spontaneous *vs* GEMM tumors). Apoptosis and angiogenesis were more variable among models (Figure 2T, 2U). Note that MVT1 scores as having very low angiogenesis despite the presence of the VEGF transgene, because blood vessels in MVT1 tumors were collapsed with no apparent lumens (Supplementary Figure 4A). Dropping the outlier MVT1 model, a significant negative correlation between angiogenesis and apoptosis was seen (Supplementary Figure 4B). None of the other parameters were significantly correlated. The models showed variable degrees of leukocytic infiltration, with leukocytes approaching 50% of the cells in the tumor for the EMT6 model (Figure 2V). There was also heterogeneity between models in the relative proportions of granulocytes (Gr1+) and T-cells (CD3+) within the tumors. In general, the spontaneous models showed a higher proportion of granulocytes in the tumor infiltrates, while the GEMM-derived models typically showed more T-cells (Supplementary Figure 4C). Using the pan-leukocyte marker CD45, distinct patterns of leukocyte distribution within and around the tumor were seen between models (Figure 2O-2R). HRM1, R3T, M6 and TSAE1 showed a relatively weak immune response overall; MET1 and F3II showed a strong immune response primarily around the tumor periphery with little infiltration into the tumor; 4T1, D2A1, MVT1 and 6DT1 showed strong infiltration of immune cells into the tumor; and E0771 and EMT6 showed massive infiltration particularly at sites of necrosis. While it is possible that these patterns of leukocyte distribution may correspond to the immunologically “ignorant”, “excluded” and “inflamed” classes that have been described for human tumors [13], more extensive immunophenotyping of leukocyte populations and activation state in the mouse tumors will be necessary to make these comparisons compelling. In general, individual tumors from the same cell line showed consistent patterns of immune cell infiltration, though the M6 model showed some heterogeneity, with occasional tumors showing a strong inflammatory response.

### Genomic characterization: single nucleotide variants

**Figure 3:**
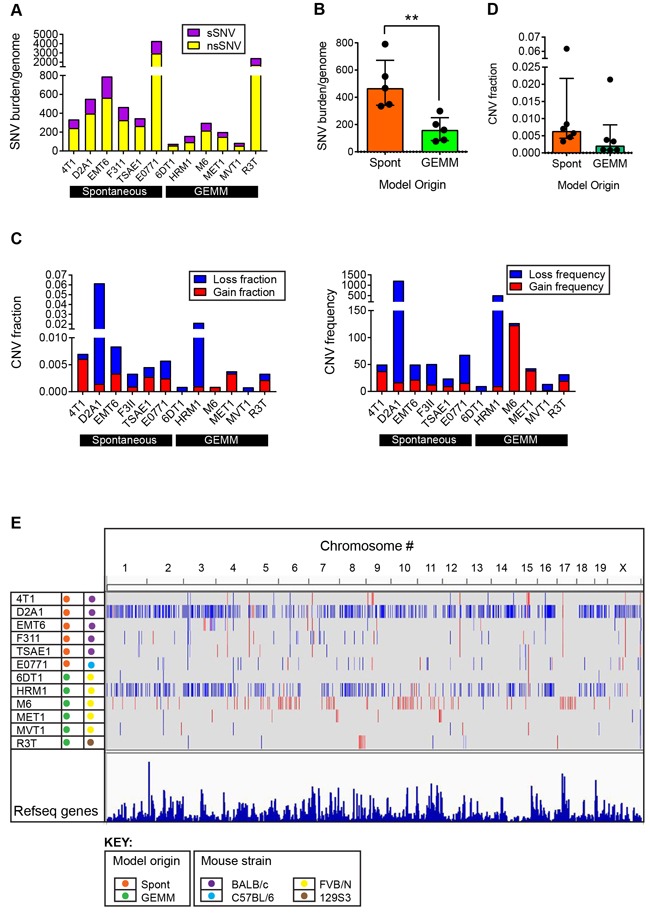
Genomic alterations in metastatic model cell lines **A**. Mutation burden in individual models. Single nucleotide variants (SNVs) in the cell lines of the model panel were identified by exome sequencing. SNVs were classified as synonymous or non-synonymous. **B**. Mutation burden (total SNVs/genome) as a function of the origin of the cell line from a spontaneous tumor (Spont) or a tumor arising in a genetically engineered mouse model (GEMM). Each point represents one model. Results are median +/− interquartile range. Mann-Whitney test. **C**. Copy number variation (CNV) in individual models. CNV loss or gain for each model is expressed as the fraction of the whole genome involved, or as the frequency across the genome. **D**. CNV burden as a function of the origin of the cell line, as in (B). **E**. Genome browser view of CNVs across entire genome for each line. Blue represents losses and red represents gains.

To assess the presence of mutations in the various tumor models, exome sequencing of gDNA from the cell lines was performed. After filtering out polymorphic variants, the majority (10/12) of the cell lines had between 50 and 800 single nucleotide variants (SNVs) in coding genes (Figure 3A). The mean ratio of non-synonymous to synonymous SNVs was 2.48 +/− 0.51 (range 1.42-3.19) when averaged across all models (see Supplementary Table 3), which is close to the ratio of ~2:1 that is seen in many human cancers [14, 15]. E0771 (C57BL/6 background) and R3T (129S3 background) were outliers with over 2000 total SNVs each. For E0771, this may reflect the long period of time since the parent tumor was originally derived in 1940 [16], with a high likelihood of spontaneous mutation and substrain drift in the intervening decades. The most frequent mutation in E0771 is A > C (Supplementary Figure 5A), which has previously been associated with oxidative stress [14]. For R3T, the high mutation burden likely relates to the 7,12-dimethylbenz[a]anthrathracene (DMBA) mutagenesis of the parent mouse [17]. Indeed, analysis of the mutational pattern of the nsSNVs shows that R3T has a very high frequency of A > T transversions (Supplementary Figure 5B), which is characteristic of DMBA-induced mutations [18, 19]. Aside from these two outlier models, among the other models, C > G transversions and C > T transitions were the most frequent events (Supplementary Figure 5C), and these are also the highest frequency alterations in human breast cancers [20], suggesting that similar mutational processes may be involved in the mouse models and in the human disease. The SNV burden/genome was significantly higher ( > 2-fold) for the models that were derived from spontaneously arising tumors than for models from genetically engineered mice (Figure 3B). This greater genetic complexity in the spontaneous models may reflect the absence of a strong driver oncogene and the longer time to development for the original tumors.

Mutation frequencies across the genome have been assessed for human breast cancer [21]. Taking the top 30 genes that are most frequently mutated in breast cancer, we assessed the occurrence of SNVs in these genes in the mouse model panel (Table 2). Details of specific mutations are given in Supplementary Table 4. 16 of the human top 30 genes have SNVs in one or more of the mouse models. *Pik3ca* mutations, which are very frequent in human breast cancer, are found spontaneously in two of the models (6DT1 and MVT1), and as a transgene in one model (HRM1). The incidence of *Tp53* mutations in the mouse panel (42%) is similar to that in human breast cancer (32%), though it should be noted that the enormous difference in size of the human and mouse datasets precludes any firm conclusions being drawn about relative mutation frequencies in the two species. The 4T1 model has previously been described to be p53 null [22], despite being genetically wildtype in our analysis. However, by Western blot, we find that the 4T1 model may be phenotypically null as the p53 protein is not detectable *in vitro* following treatment with a DNA-damaging agent (Supplementary Fig 6). The most frequently mutated gene in the mouse panel is *K-Ras* which is present in 5/12 models, including both spontaneous and GEMM-derived lines. This mutation is relatively rare in human breast cancer ( < 2% of cases in most studies). However, activation of the ras/MAPK pathway is seen frequently, particularly in triple negative breast cancer [23], and *KRAS*G12D was recently shown to induce tumorigenesis of normal human breast epithelial cells with high efficiency as a single oncogenic event [24].

**Table 2:** Single nucleotide variation incidence in mouse model panel for top 30 genes most frequently mutated in human breast cancer

		Mouse model													
4T1	6DT1	D2A1	E0771	EMT6	F3II	HRM1	M6	MET1	MVT1	R3T	TSAE1	Mutation rate in human BrCa (%)	Mutation rate in mouse panel (%)
TCGA mutated gene	*Pik3ca*	0	**1**	0	0	0	0	2*	0	0	**1**	0	0	32.6	25
	*Trp53*	0†	0	0	**2**	0	**1**	0	0**	**2**	0	**2**	**2**	31.5	41.7
	*Ncor1*	0	0	0	0	0	0	0	0	**1**	0	0	0	3.8	8.3
	*Map2k4*	0	0	0	**1**	0	0	0	0	0	0	0	0	3.7	8.3
	*Pten*	0	0	0	0	**2**	0	0	0	0	0	0	0	3.5	8.3
	*Akt1*	0	0	0	0	**1**	0	0	0	0	0	0	0	2.1	8.3
	*Spen*	0	0	**2**	**2**	**1**	0	0	0	0	0	0	0	2	25
	*Tbx3*	0	0	0	0	0	0	0	0	**1**	0	0	0	2	8.3
	*Sf3b1*	0	0	**1**	0	0	0	0	0	0	0	0	0	1.9	8.3
	*Arid1a*	0	0	0	0	**1**	0	**1**	0	0	0	0	0	1.9	16.7
	*Erbb2*	0	0	0	0	0	0	0	0	**1**	0	0	0	1.7	8.3
	*Med23*	0	0	0	**1**	0	0	0	0	0	0	0	0	1.6	8.3
	*Tbl1xr1*	0	0	**1**	**2**	0	0	0	0	0	0	0	0	1.1	16.7
	*Casp8*	0	0	0	0	0	0	0	0	0	0	**1**	0	1.1	8.3
	*Cul4b*	0	0	0	0	0	0	0	0	**1**	0	0	0	0.8	8.3
	*Kras*	0	**2**	0	**2**	0	0	**2**	0	0	**2**	0	**2**	0.7	41.7

Mutations were identified by exome gDNA sequencing of cell lines. Gene mutation status is given by 0 (wildtype), 1 (heterozygous) and 2 (homozygous). Details of individual mutations are given in Supplementary Table 4. The top 30 most frequently mutated genes in human breast cancer were taken from Lawrence et al [21]. Genes in the top 30 with no mutations in the panel were *Gata3, Map3k1, Mll3, Cdh1, Runx1, Pik3r1, Ctcf, Cbfp, Tbx3, Foxa1, Rb1, Mll, Stag2, Myb, Hist1h3b, Cdkn1b, Rab40a*. *Activating mutation is in the human transgene. †p53 is genetically wildtype but protein is undetectable. **p53 is functionally inactivated in this model by the SV40 T transgene.

### Genomic characterization: copy number variants

**Figure 4:**
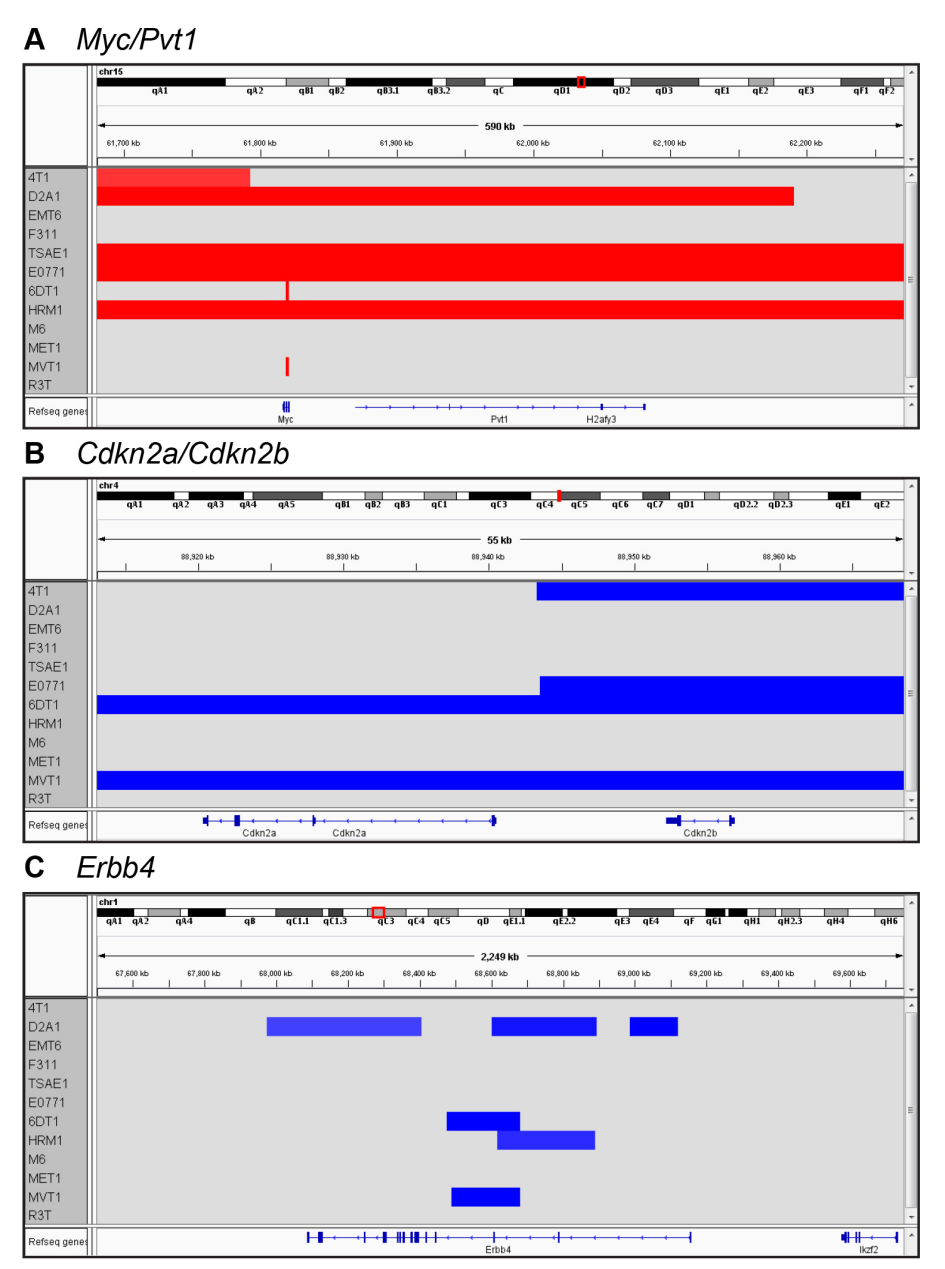
Recurrent local amplifications and deletions in the metastatic models Genome browser view of CNVs in the vicinity of the *Myc/Pvt1* locus **A**., the *Cdkn2a/Cdkn2b* locus **B**., and the *Erbb4* locus **C**.

Copy number variation (CNV) across the mouse genome was assessed using the Affymetrix^®^ Mouse Diversity Genotyping array. The genome-wide burden of losses and gains varied widely across the model panel (Figure 3C), with a trend to a higher CNV fraction in the spontaneous models (Figure 3D). HRM1 and D2A1 showed a relatively high copy number loss fraction and frequency, while M6 had a high frequency of small copy number gains (Figure 3C, 3E). Among the genes commonly amplified or deleted in human breast cancer, only amplifications in *Myc* and *Mdm2* and deletions of *Cdkna, Cdkn4b, Csmd1* and *Ptprd* were seen in the mouse tumor panel (Supplementary Table 5). 6DT1 and MVT1 showed focal amplifications of *Myc*, reflecting the presence of the MMTV-Myc transgene in these models. D2A1, E0771, HRM1 and TSAE1 all had larger amplicons that included the entire *Myc* locus, and extended to include the adjacent *Pvt1* locus (Figure 4A). Approximately 10% of all human breast cancers show co-amplification of *MYC* and *PVT1,* with a particularly high proportion (62%) among HER2+ tumors [25]*. Pvt1* encodes a long non-coding RNA that stabilizes Myc protein expression, and low copy number gains in *Myc* and *Pvt1* cooperate to promote breast cancer development in mouse models [25]. *Cdkn2b* was homozygously deleted in 4T1 and E0771, while in 6DT1 and MVT1, the region of deletion extended to include *Cdkn2a* as well as *Cdkn2b* Figure 4B). Deletions in these cyclin-dependent kinase inhibitors are found in ~4% of human breast cancers [26]. Few other recurrent amplifications or deletions were seen across the panel. The *HER2/ERBB2/Neu* locus, which is a frequent site of amplification in human breast cancers, was not amplified in any models. However the *Erbb4* locus on chromosome 1qC3 showed recurrent internal focal deletions encompassing exons 2 and/or 3 in four of the models (6DT1, D2A1, HRM1 and MVT1) (Figure 4C). ERBB4/HER4 is the receptor for the Neuregulin family of ligands, with conflicting reported roles in breast cancer that may depend on the splice variant considered [27] [28]. Oncogenic gain-of-function mutations in the extracellular domain of ERBB4 that increase ligand-independent receptor activation have been described in melanoma [29], and it will be interesting to determine whether the focal deletions in the ERBB4 extracellular domain in the mouse models might have a similar effect.

### Transcriptomic architecture of mouse model panel

**Figure 5:**
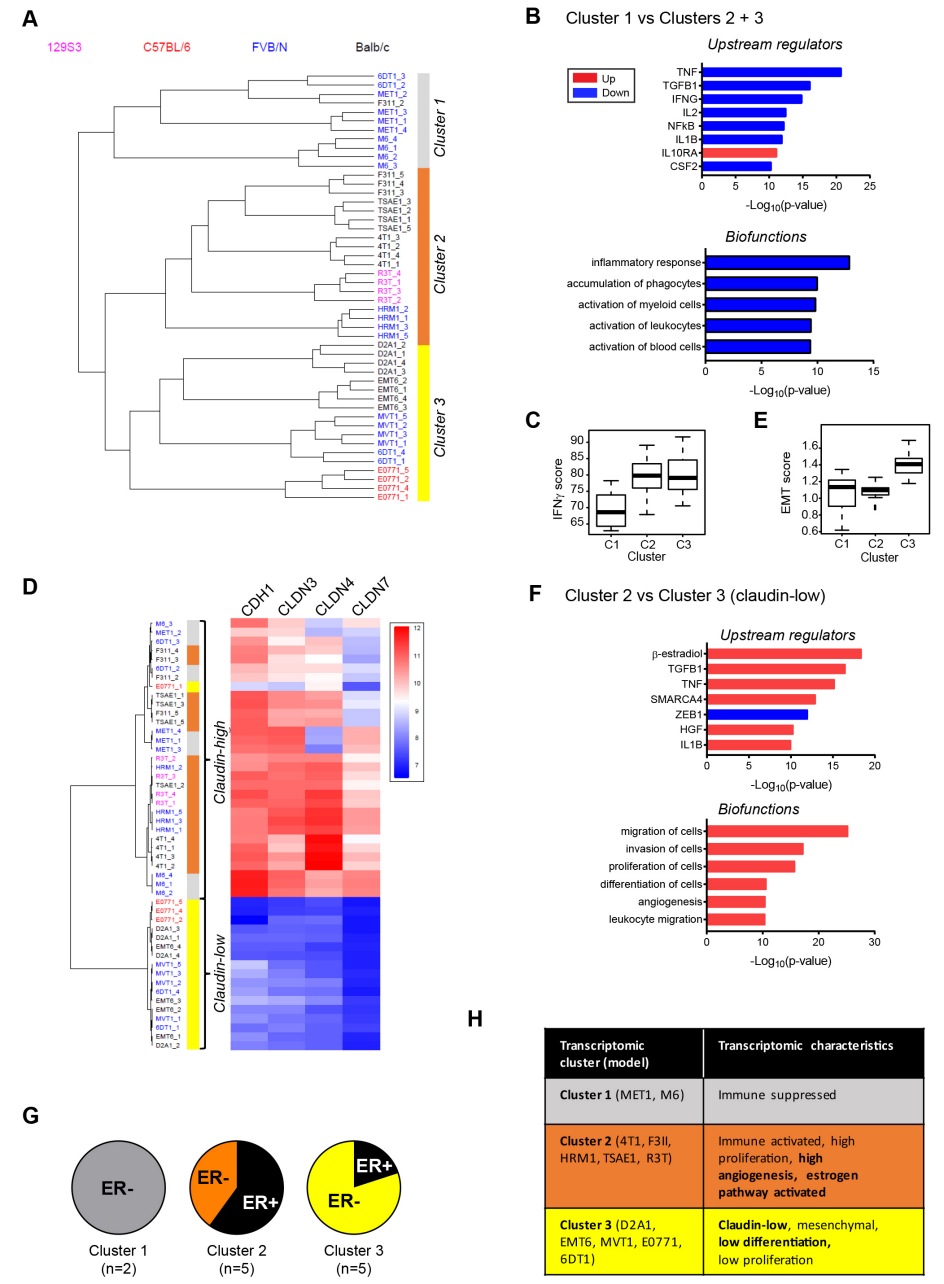
Transcriptomic architecture of the mouse tumor panel **A**. Unsupervised hierarchical clustering of transcriptomes from primary tumors of mouse model panel for 4 tumors/model identifies 3 distinct clusters. **B**. Biofunction and upstream regulator enrichment analysis in differentially expressed genes from cluster 1 *vs* clusters 2 and 3. Blue indicates downregulated in cluster 1 and red indicates upregulated. **C**. The interferon-γ (IFNg) gene signature score is lowest in cluster 1 tumors. C2 *vs* C1, *p* < 0.001; C3 *vs* C2, ns; C3 *vs* C1, *p* < 10e-04 **D**. Cluster 3 could be segregated from the other two clusters based just on the low expression of E-cadherin and three claudins. Cluster 3 represents the “claudin-low” phenotype. **E**. The EMT gene signature score is highest in cluster 3 tumors. C2 *vs* C1, ns; C3 *vs* C2, *p* < 10e-08; C3 *vs* C1, *p* < 0.001. **F**. Biofunction and upstream regulator enrichment analysis of differentially expressed genes from cluster 2 *vs* cluster 3. Blue indicates downregulated in cluster 2 and red indicates upregulated. **G**. Fraction of tumor models in each cluster that are ER+ by immunohistochemistry. **H**. Summary of characteristic properties of the different model clusters predicted from transcriptomic analyses. Properties in bold were validated by orthogonal techniques (immunohistochemistry or histopathology).

To probe more of the tumor biology captured in the model panel, the transcriptomes of four orthotopically-implanted primary tumors from each of the 12 models were analyzed using the Affymetrix^®^ Mouse Gene 1.0ST array. Unsupervised hierarchical clustering showed that the mouse models fell into three main transcriptomic clusters (Figure 5A). Models from different mouse strains were distributed between the three clusters, indicating that genetic background was not the major driver of the transcriptomic differences. Cluster 1 (primarily M6 and MET1) was characterized most notably by low expression of chemokines and immune modulators (eg. CCL2, CCL13, CCL7, CXCL3, CXCL10, CXCL16, CSF1, IL1, IL18, IL24) and indications of low cytotoxic T-cell function (low granzyme B). IPA^®^ upstream regulator analysis of differentially expressed genes predicts that the immunosuppressive cytokine IL10 is active in the Cluster 1 tumors, while activity of pro-inflammatory cytokines is suppressed (Figure 5B). IPA^®^ biofunction analysis further predicts that Cluster 1 tumors are relatively immunologically silent, with decreased inflammation, immune cell recruitment and activation in these models (Figure 5B). Similarly, a core interferon-γ gene signature that is associated with response to immune checkpoint inhibitors has much lower score in this cluster (Figure 5C; Supplementary Figure 7A for individual models). As noted earlier, immunostaining for the pan-leukocyte marker CD45 showed little leukocyte infiltration into the M6 tumors, and leukocytes predominantly localized round the tumor margins for MET1 tumors.

Cluster 3 tumors (D2A1, EMT6, MVT1, E0771, 6DT1) were strikingly characterized by very low expression of claudin genes. Indeed, models in cluster 3 could largely be segregated based just on the expression of E-cadherin and three claudins (Figure 5D). A “claudin-low” subtype of human breast cancers was originally identified in gene expression analysis of combined mouse and human mammary tumor datasets, and was subsequently shown to capture a poor prognosis, metaplastic cancer subtype with features of mammary stem cells [30]. The tumor models in cluster 3 were also predicted to be claudin-low using a published human 1390-gene claudin-low predictor [31] (data not shown). Consistent with their claudin-low status, the Cluster 3 tumors had a high score for a consensus gene signature that defines cancer-associated epithelial to mesenchymal transition (EMT) (Figure 5E; Supplementary Fig 7B for individual models). The 6DT1 model split between Clusters 1 and 3, but all four tumors had a high EMT score so the model is probably claudin-low.

In cluster 2 tumors (F311, TSAE1, 4T1, R3T, HRM1), β-estradiol was predicted to be the most highly activated upstream regulator (Figure 5F), consistent with enrichment of models that were immunohistochemically ER-positive (Figure 5G). Proinflammatory cytokines such as TNF and IL1B were also predicted to be activated. Thus, although cluster 2 tumors have lower total leukocyte numbers than cluster 3 tumors as assessed by immunohistochemistry (Supplementary Figure 7C)), the leukocytes in cluster 2 tumors may be more highly activated, or these cytokine pathways may be activated in the tumor cells themselves. It will be important in the future to do comprehensive FACS-based immunophenotyping to get a more detailed assessment of functional leukocyte subtypes and their activation status. As expected ZEB1, a key regulator of EMT, was less active in cluster 2 than cluster 3 (claudin-low) tumors. Analysis for enriched biofunctions suggested that cluster 2 tumors would be more invasive and proliferative than the cluster 3 claudin-low tumors, as well as more differentiated and more angiogenic (Figure 5F). Significantly higher angiogenesis in cluster 2 tumors was confirmed by immunohistochemistry (Supplementary Figure 7D). The lower proliferation signal in cluster 3 tumors was not seen at the immunohistochemical level (Supplementary Figure 7E), but is consistent with data suggesting that the claudin-low human breast cancers lack a strong proliferation signature [30]. The lower invasion/migration signal in the claudin-low cluster 3 tumors is unexpected and needs further investigation. Key transcriptomic features of the three clusters are summarized in Figure 5H.

### Optimization of subtype assignment strategies for mouse models

Transcriptomes of human breast cancer have been classified into 5 intrinsic subtypes with prognostic significance [32]. To determine which mouse allograft models map to which intrinsic human subtypes, seven different computational methods were evaluated using four different gene lists as detailed in Methods, to identify the optimal combination for subtype calling across species. The results of the analysis indicated that the clustering method was the most robust at subtype calling, as measured by Area Under the Curve (AUC) analysis (Supplementary Table 6), followed by the Principle Component Analysis (PCA) method. All four of the method/gene list combinations that involved clustering as the computational method gave high AUC scores, with the clustering method/G1841 genelist combination being best overall. When applied to the mouse models, the seven computational methods were similar in their classification of the majority (11/12) of models as non-basal, but there were differences in their assignment of models to the luminal B and HER2-enriched classes (see Supplementary Table 7 for results with the G1841 list). In relation to optimal genelists for the analysis, the distribution and robustness of subtype calls for the mouse (G1841) and human (G1918) intrinsic gene lists were highly similar (Supplementary Table 8) demonstrating that the mouse and human gene lists were approximately equal in their ability to discriminate human breast cancer subtypes.

### Intrinsic subtype assignment of mouse allograft models

**Figure 6:**
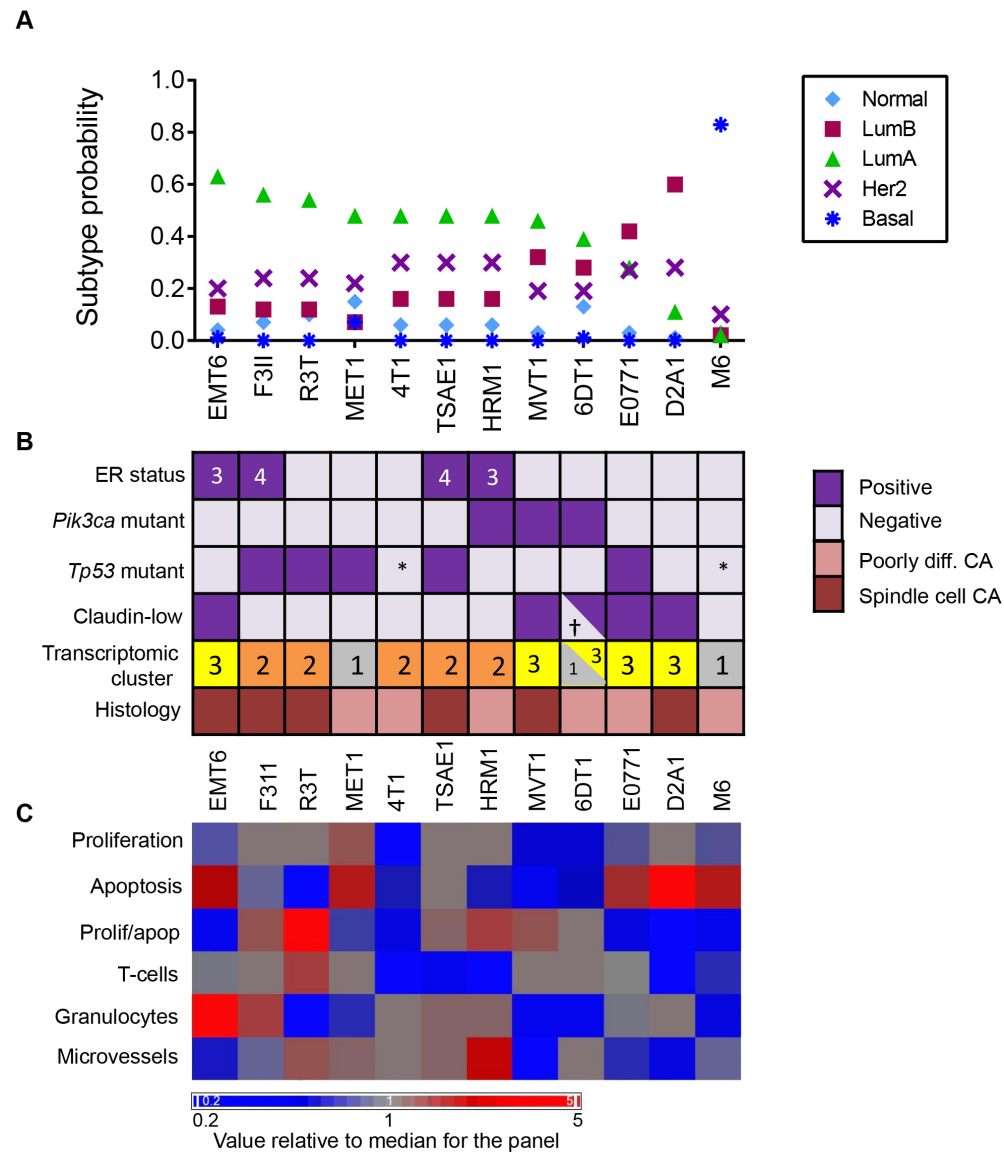
Intrinsic Subtypes and Related Biological Properties of Primary Tumors from the Mouse Model Panel **A**. Subtype call probabilities for orthotopic tumors were generated from the tumor transcriptomic datasets using the G1841 gene list and cluster method as described in Methods. Results are mean values for 4 tumors/model. Models are ordered by decreasing luminal A component. **B**. Molecular and histopathological features of the models, ordered as in A. ER status was determined by IHC and the numbers in the boxes represent the Allred score. *Pik3ca* and *Tp53* mutation status were assessed by exome sequencing. *Functionally p53 null. Claudin-low status was determined using the transcriptomic Claudin-low predictor. †2/4 tumors were called as claudin-low. Cluster number refers to the transcriptomic subtype from Figure 5, with cluster 3 being the claudin-low cluster. Histopathological diagnosis uses human nomenclature. **C**. Proliferation, apoptosis, immune cell infiltration and angiogenesis indices were assessed quantitatively by immunohistochemistry and the mean value for 3 tumors/model was determined. Mean values for each model were then median-centered across the model panel for heatmap generation. Models are ordered as in A.

Using the clustering method with the G1841 mouse intrinsic genelist, the majority of the mouse allograft models were assigned to the Luminal A class (Figure 6A). Exceptions were E0771 and D2A1 which were predominantly luminal B. Only the M6 model, derived from the C3(1)TAg transgenic mouse, was transcriptomically basal. Using the much smaller PAM50 genelist with the Cluster method, again only M6 was classified as basal, though some of the other models showed a higher luminal B component with this genelist, and HRM1 was classified as predominantly HER2 enriched (Supplementary Figure 8). The assignment of M6 to the basal subclass is consistent with published reports that tumors from the parental C3(1)TAg transgenic model are also classified as basal [33, 34].

The assignment of the other models to the luminal subclasses was more unexpected so we looked at other properties that are expected to correlate with the luminal subtype. Despite a uniformly aggressive and poorly differentiated or spindled histopathology, four of the nine models assigned to the luminal A subclass were weakly ER-positive (Figure 6B), consistent with observations that the majority of ER+ human breast cancers fall into luminal A intrinsic subtype [30]. Similarly, three of the nine luminal A mouse models had *Pik3ca* mutations, which are also enriched in human luminal A tumors [35]. An MMTV-Pik3ca-H1047R transgenic model was previously described to be ER+ [36] and with a luminal expression profile [33]. TP53 mutations are found at very high frequency (80%) in human basal breast cancer, but they are also seen in a significant fraction of human luminal A (12%) and luminal B (29%) tumors, so the presence of Tp53 mutations in 5 of the 11 models that were classified as either luminal A or B is at higher incidence than seen in the human counterparts, but not entirely implausible. MVT1 and 6DT1 were both derived from transgenic models that included the MMTV-Myc transgene and these models were subtyped as luminal, but could not be unambiguously assigned to luminal A vs luminal B. The parental MMTV-Myc transgenic model was linked with both basal-like and luminal B subtypes in a previous study ([33]. Human basal breast cancers are characterized by a proliferation gene cluster and a more aggressive biology [30]. We did not see a clear correlation between intrinsic subtype in the mouse models and biological parameters that might associate with more aggressive disease (Figure 6C). If anything, there was a trend toward increased apoptosis and reduced immune cell infiltration and reduced microvessel density in the luminal B and basal mouse tumors compared with the luminal A tumors. However, it should be emphasized that this is a small dataset and that the mouse models were pre-selected for clinically aggressive metastatic disease.

### Relatedness to human patient derived xenografts

**Figure 7:**
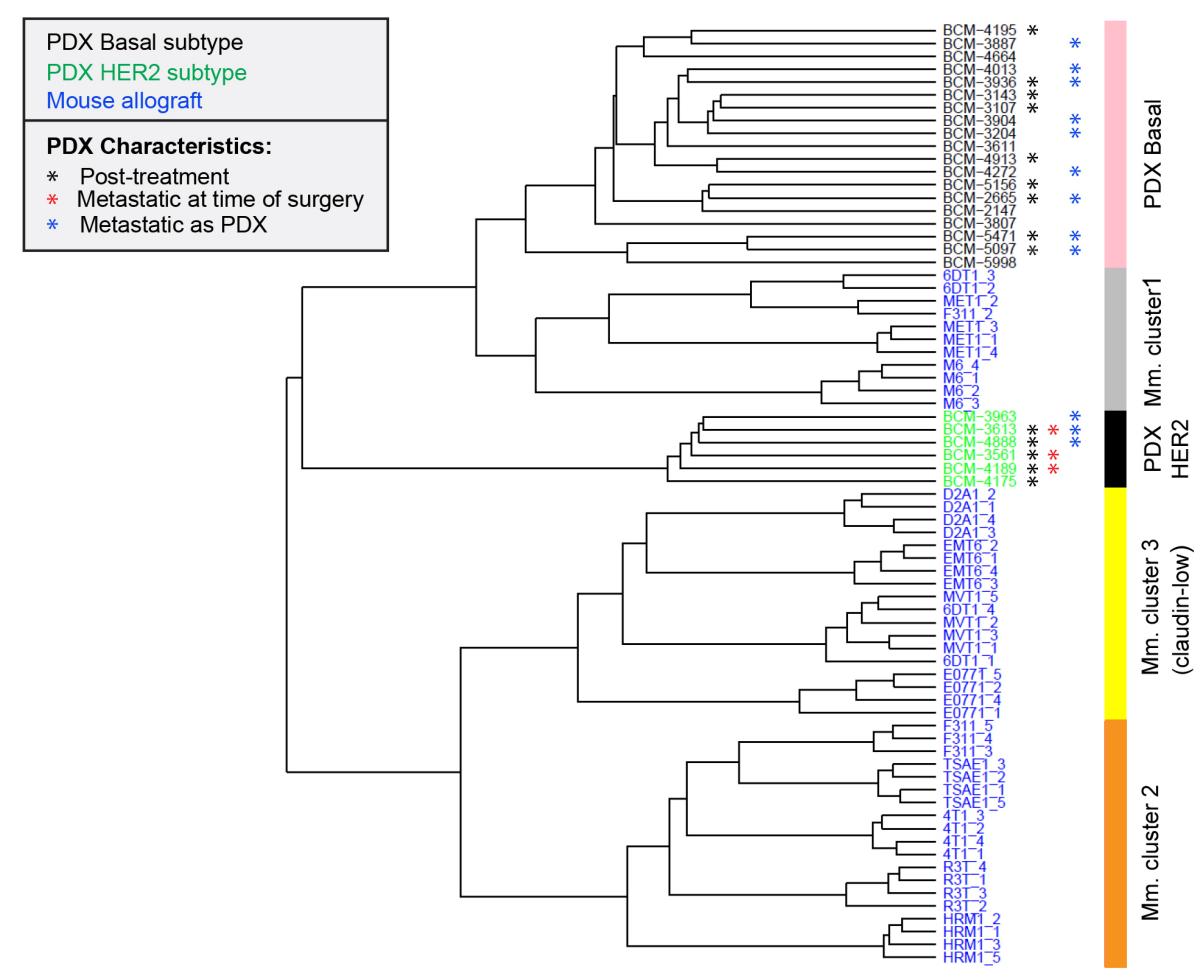
Relationship of mouse tumor allografts to human patient-derived xenografts Unsupervised hierarchical clustering of mouse model (Mm) primary tumor transcriptomes with transcriptomes of human breast cancer-derived xenografts (PDX) Metastasis-relevant characteristics of the PDXs are indicated. Post-treatment indicates that the PDX was established from a patient who had received systemic neoadjuvant therapy.

Human patient-derived xenografts (PDXs) are increasingly being explored as useful avatars of human disease [37]. We assessed the transcriptomic relatedness of the immunocompetent mouse allograft models to a published panel of 25 human breast cancer PDXs [38]. After normalization and batch effect removal, unsupervised hierarchical clustering of the merged datasets showed that mouse model cluster 1 (predominantly M6 and MET1) clustered in the same arm of the dendrogram as the human PDXs while mouse cluster 2 and cluster 3 were distinct (Figure 7). The mouse cluster 1 allografts were more closely related to the human basal PDXs than the human HER2 PDXs, which occupied a separate arm on the dendrogram. No luminal PDXs were represented in this dataset. The biological basis of the transcriptomic relatedness of the mouse cluster 1 models to the human PDXs needs to be further explored, but could in part reflect the observation that the cluster 1 models mouse are predicted to be relatively immunologically silent, and the human PDXs were implanted in immunodeficient mouse hosts.

## DISCUSSION

Human breast cancer is a heterogenous disease [39]. Considerable variation in clinical course, histopathology, genetic characteristics and transcriptomic profiles is seen between patients, and disease within a given patient can also show locoregional and chronological variation. Metastasis is the lethal end-stage of cancer progression, and few if any effective treatments exist for established metastatic disease [1]. One reason for this failure is the paucity of preclinical drug development studies that use metastatic burden as an endpoint. Another is the reliance on a very small number of metastatic models that may lack predictive power because they capture too little of the heterogeneity of the human disease. To address these issues, we have characterized a panel of 12 cell line-based mouse allograft models of metastatic breast cancer. Conditions were established in which the models metastasize to the lungs with a frequency at least 50%, and in most cases metastasis incidence rates of 80-100% were achieved. Further optimization is still possible, and the use of alternative delivery routes (eg. intracardiac injection) could expand the range of metastatic sites to include bone, brain and viscera.

### Heterogeneity captured by the model panel

The allograft panel captures at least some of the heterogeneity seen in human breast cancer. At a genetic level, over half of the 30 most frequent mutation events in human breast cancer and several of the most frequent copy number variation events are represented in the panel. *Kras* mutations, *Myc* amplification and *Cdkn2a/b* deletions are somewhat over-represented when compared with their incidence in human breast cancer. Since frequent *MYC* amplification and *CDKN2A/B* deletions were also seen in a recently established bank of human breast cancer PDXs [40], these genetic events may contribute to efficient engraftment. In addition to the range of somatic tumor mutations found in the panel, the use of models from four different mouse strains also contributes some of germline genetic variation seen in humans. Histologically the models all formed poorly differentiated or sarcomatoid/spindled tumors at the primary site, which are not common histologies in human breast cancer. However, there was cross-panel heterogeneity in many other clinically relevant biological properties, including proliferative index, angiogenesis, and patterns of leucocyte infiltration into the primary tumors. The transcriptomic data suggested that the models may fall into immunologically active (cluster 2 models) and immunologically suppressed (clusters 1 and 3 models). It will be of interest to see if these correspond to the “T-cell inflamed” *vs* “non-T-cell inflamed” human tumor classes that correlate with response to immunotherapy [41]. This immunologic heterogeneity may be useful in the development of immune-based therapies for metastasis.

### Classification of the models in relation to human disease subtypes

Multiple different strategies for sub-classifying human breast cancer are currently in use. All emphasize different types of information, which may or may not be directly interrelated. The simplest molecular classification used to guide therapy involves three markers, namely estrogen receptor (ER), progesterone receptor (PR), and HER2 amplification status. Using that classification scheme, the majority (8/12) of the mouse models in the panel are “triple negative” for all three markers. Four of the models (EMT6, F311, TSAE1 and HRM1) were weakly ER-positive by immunohistochemistry and all of these except EMT6 fell into a transcriptomic cluster (Cluster 2) that had predicted activation of estrogen as an upstream regulator. Since the mouse GEMM models of breast cancer are generally ER-negative [34], the presence of ER-positive models in the allograft panel is a potential strength. However, currently it is not known whether the allograft models are hormone-dependent for tumor growth or metastasis.

More recently, human breast cancers have been classified into different molecular subtypes on the basis of transcriptomic characteristics [42], with or without additional genomic information [43]. The most widely-used transcriptomic classification identifies 5 distinct “intrinsic” subtypes (luminal A, luminal B, HER2-like, normal-like and basal). Applying this classification scheme to the mouse model panel, the majority of the models were found to be luminal A or luminal B. Only a single model (M6) classified as basal, which was consistent with the basal classification of the parental C3(1)Tag GEM model from which the M6 cell line was derived [34]. The transcriptomic classification of most of the allograft models as luminal A or B was unexpected, given their aggressive histopathology and clinical behavior, and the low or absent hormone receptor expression. However, this class assignment was robustly seen with a variety of different classifier genesets and algorithms, and was consistent with the ER-positivity of four of the models, and the presence of *Pik3ca* mutations in another two. There are human/mouse differences in hormone receptor expression in the normal mammary gland, including much lower ERa expression in mouse luminal progenitors [44]. This may lead to mouse tumors that are transcriptomically luminal, but nevertheless are “triple negative” by marker analysis. Indeed, it has been suggested that the human “luminal” transcriptome has two components, one driven by ER and the other by GATA3, and that the ER-driven component is largely missing in mouse tumors [34]. Thus the “triple negative” designation in mouse may not encompass the same biology as it does in human.

Finally, using an orthogonal transcriptomic classifier, five of the luminal models were found to also be “claudin-low” (EMT6, MVT1, 6DT1, E0771, D2A1). The claudin-low signature is associated with a relatively rare metaplastic subtype of human breast cancer, and has hallmarks of a stem-like, epithelial-to-mesenchymal transition state [30]. This signature is enriched in human breast cancers following endocrine therapy or chemotherapy [45], so the claudin-low tumors may be useful as models of recurrent disease. The claudin-low phenotype is also over-represented in human breast cancer cell lines [46], suggesting it may confer a selective advantage in tissue culture.

### Relationship of mouse models to PDXs

Patient-derived tumor xenografts are an emerging tool for drug development or selection studies. They largely recapitulate the histological and transcriptomic characteristics of the original tumor [40], and to the extent that it has been tested, the response of the PDX to drugs correlates well with clinical outcome when the patient is treated with a drug selected through a PDX screen [47]. Thus there is hope that PDXs will enable precision medicine approaches to cancer therapy. Realistically, the routine generation of PDXs from every individual patient is likely to be cost- and time-prohibitive for guiding therapy selection in real time. It will be more feasible to map the tumor from any given patient onto a closely-related avatar from an established panel of representative PDXs for which the response to different therapeutic agents/strategies has already been assessed [37]. However, one key limitation of these PDXs currently is the lack of an immune component. Here we addressed whether it would be possible to find mouse avatars of the human avatars by comparing the transcriptomes of the mouse panel tumors with a panel of human breast cancer PDXs. Interestingly our Cluster 1 mouse models (MET1, M6 and some 6DT1) cluster in with the human PDXs, suggesting that with an expanded panel of mouse allograft models, this approach might be feasible.

### How to use the models

All models have strengths and limitations that need to be recognized so that they can be used optimally. The advantages of the mouse allograft models include that they are fully immunocompetent, there is no species incompatibility in paracrine interactions between tumor and stroma, the disease course is rapid, and metastasis burden can realistically be used as an endpoint in therapeutic intervention studies. On the negative side, the histology of the primary mouse allograft tumors is unlike that of most human breast cancers, the rapid time course may skew the biology, and the use of established cell lines may introduce bias due to selection of properties that allow propagation on plastic. However, we would argue that for the development of anti-metastatic therapies, the robust metastatic phenotype and the presence of an intact immune system trumps the other limitations of these models for now. In the future, further advances may come from the use of never-on-plastic mouse allograft models [48, 49] or metastatic PDXs in mice with reconstituted human immune systems [37]. It should also be fully appreciated that there will be no simple one-to-one mapping of any mouse model onto human cancer subtypes with respect to every characteristic. The mapping is multidimensional, with different results in different dimensions (genetic, transcriptomic, histopathology, clinical behavior etc). Model selection should be driven by which aspect of the biology is most important for the question being asked.

In summary, we have extensively characterized a panel of metastatic mouse allograft models of breast cancer that captures some of the heterogeneity of the human disease. This panel should serve as a useful platform for anti-metastatic drug screening and predictive biomarker development.

## MATERIALS AND METHODS

### Ethics statement

All animal studies were conducted under protocol LC-070 approved by the Animal Care and Use Committee of the National Cancer Institute, The Frederick National Laboratory and the Center for Cancer Research are accredited by AALAC International and follow the Public Health Service Policy for the Care and Use of Laboratory Animals. Animal care was provided in accordance with the procedures outline in the “Guide for Care and Use of Laboratory Animals” (National Research Council; 2011 National Academies Press; Washington, DC).

### Model acquisition and cell culture

Metastatic murine mammary cancer cell lines were obtained from the originating laboratories and/or investigators as detailed in Table 1. Cell lines were maintained in culture using growth media and optimal split ratios as indicated in Supplementary Table 9. Care was taken not to use very high split ratios and not to let the cells go confluent at any time. All lines were tested and shown to be free of mouse viral pathogens and mycoplasma. To assess effects of estrogen on tumor cell growth *in vitro*, tumor cells were seeded at 20,000 cells/well in 24 well plates in their normal growth medium but using phenol red-free medium and charcoal-stripped serum supplemented with 10ng/ml EGF and 10μg/ml insulin, and were treated with 17-β-estradiol (Sigma E2758) at a final concentration of 100nM, or vehicle control. Cell proliferation over 42h was assessed by measuring culture confluence using an IncuCyte ZOOM^®^ Live Cell Analysis System (Essen Biosciences).

### Animal studies and tissue collection

Pilot experiments using different cell innocula, different experimental formats and different time-frames were run to optimize the metastatic efficiency for each model. Ideally we wished to find conditions that gave lung metastases in at least 40% of the mice on study following orthotopic implantation of the primary tumors, with surgical resection when tumors reached 5-8mm diameter, and metastatic burden being detectable within 60 days of tumor cell implantation. The detailed experimental conditions used to generate metastases from all these models in our facility are given in Supplementary Table 1. However, note that there is room for further optimization and that results are likely to be facility-dependent. The #4 mammary fat pad (mfp) was the preferred site for tumor cell implantation because primary tumor resection with clean margins is easier at this site and tumors can grow to a larger size without impairing animal mobility. The fatpad was surgically exposed to ensure that cells were actually implanted into the mfp rather than in the vicinity of it. If metastatic efficiency was too low from the #4 mfp, the #2 mfp was tried as this generally gives a higher frequency of metastases, but the surgery is more complex because of the high vascularity of tumors at this site and there is then a higher risk of local recurrence and post-operative complications. For the EMT6 model, cells were implanted in a 1:1 mix with reduced growth factor Matrigel (BD Biosciences). If metastatic frequency following tumor resection was low, the model was run without resection (E0771, MVT1, R3T). If metastatic frequency was still undesirably low following orthotopic implantation, cells were introduced into the mice via the tail vein (F3II, MET1 and TSAE1). At the experimental endpoint, mice were euthanized by carbon dioxide narcosis followed by thoracotomy. Primary tumors were bisected and snap frozen in liquid nitrogen for molecular analyses, or fixed in 10% neutral buffered formalin (NBF). Lungs were inflated with 10% NBF and fixed, and the individual lung lobes were separated prior to embedding in a single block. Fixed tissues were paraffin embedded for histology and metastases were enumerated on an H&E-stained cross-section of all lung lobes. For the chemotherapy intervention study, mice were implanted with 200,000 MVT1 cells in the #4 mammary fat pad, and three once-weekly injections of cytoxan (10mg/Kg i.p. in PBS) were given to each animal starting when tumors became palpable (3mm diameter). A control cohort received injections of PBS.

### Histopathology and immunohistochemistry

Histopathology. Hemotoxylin and eosin (H&E) stained sections of three representative primary tumors for each tumor model were assessed initially by one veterinary pathologist (MRA) and a consensus diagnosis was achieved through a pathology slide conference with two additional veterinary pathologists (DCH and PM), including information from slides immunostained for additional diagnostic markers, including wide-spectrum cytokeratin, cytokeratin 8, α-smooth muscle actin and vimentin. Details of the antibodies used for all immunohistochemistry and the immunostaining conditions are summarized in Supplementary Table 10. A human anatomic pathologist (PHW) contributed diagnoses from a human pathology perspective.

Hormone receptor status. To determine estrogen receptor and progesterone receptor status, immunostained sections of three representative primary tumors from each model were assessed independently by a veterinary pathologist (MRA) and a surgical pathologist (PHW). Where results were discrepant, additional tumors were examined for discrepant models (F311 and HRM1) and a consensus was reached. ER status was determined in parallel with adjacent sections stained for cytokeratin 8 (CK8) since mouse mammary stromal cells are estrogen receptor positive [11] and in many of the models, the tumor cells had a spindled morphology. Parallel CK staining, cell morphology and growth patterns allowed positive tumor cells to be distinguished from positive stromal cells. The extent of ER positivity in tumors was scored by the surgical pathologist using the Allred scale [50], which scores the % of cells stained on a scale of 0 to 5 and the intensity of staining on a scale of 0 to 3, for a maximum possible total score of 8.

Additional immunohistochemical markers. Three representative primary tumors for each model were immunostained for Ki67 (proliferation), Caspase3 (apoptosis), CD34 (angiogenesis), CD45 (pan-leukocyte), CD3 (T-cell), Ly6G (granulocyte) markers. Details of the antibodies and conditions used for immunostaining are given in Supplementary Table 10. All immunostaining steps from deparaffinization through counterstaining were performed using a BondMax Autostainer (Leica Biosystems). Immunostained slides were scanned at 20x magnification using an Aperio Scanner. Images of the entire tumor section were manually segmented to exclude regions of intra-tumoral necrosis and of stroma surrounding the tumor, and automated Aperio-designed algorithms were run to assess the positive cells as % total nuclei in the segmented region (Ki67, caspase3 and leukocyte markers) or microvessel density (CD34: # microvessels/μm^2^).

### Western blot analysis for p53 and p21

Cultured cells were treated with 0.5μM Adriamycin for 6 hours to induce p53. Western blots of the cell lysates were run to assess expression of p53 and the canonical p53 target p21. Tubulin was the loading control. Antibodies used were p53, sc-1312, Santa Cruz Biotechnology; p21, sc397, Santa Cruz Biotechnology; α-tubulin, T6199, Sigma-Aldrich.

### Genomic characterization of tumor cell lines

Copy number variant (CNV) analysis. RNA-free genomic DNA (gDNA) was prepared from each cultured cell line using the Gentra Puregene Cell Kit (Qiagen) according to manufacturer's protocol and resuspended in low EDTA TE buffer. DNA was labeled using the Affymetrix^®^ SNP 6.0 protocol and hybridized to the Affymetrix^®^ Mouse Diversity Genotyping array, which interrogates more than 623,000 single nucleotide polymorphisms (SNPs) and 916,000 non-polymorphic regions across the genome, allowing mapping to a resolution of 4.3 kb. Arrays were scanned using the Affymetrix GeneChip 3000 7G Plus scanner. Images and intensity data were collected using the Affymetrix GeneChip Command Console (AGCC) software, and data were analyzed using the R package MouseDivGeno [51]. Data from normal tissue of matched mouse strains (http://cgd.jax.org/datasets/diversityarray/CELfiles.shtml) were used as references to identify strain-specific regions of copy number gain or loss, which were then removed from the processed datasets. CNV data have been deposited in GEO under accession # GSE69902. CNV fraction was calculated by adding up genomic regions affect by CNV loss or gain and dividing by the total genome size. CNV frequency was calculated by counting the number of genomic regions affected by CNV loss or gain.

Single nucleotide variant (SNV) analysis. gDNA was prepared as for CNV analysis. Exome libraries were prepared using the Agilent SureSelect Mouse all-Exon target enrichment kit. Deep sequencing was performed on a HiSeq 2000 with TruSeq V3 chemistry, multiplexing of 3 samples/lane. Mean target coverage depth was 85-141X with 84% of regions having > 30x coverage. Samtools mpileup was used to call sequence variants for the 12 cell lines with mm10 as the reference genome. Mouse strain-specific single nucleotide polymorphisms (SNPs) were filtered out using dbSNP137 and the Sanger database for variants identified from whole genome sequencing of 17 mouse strains (ftp://ftpmouse.sanger.ac.uk/current_snps/mgp.v3.snps.rsIDdbSNPv137.vcf.gz). Variants after removing dbSNP137 and mgpSNPs were annotated with ANNOVAR (http://annovar.openbioinformatics.org/en/latest) [52]. Exonic SNPs were identified and variants were classified as synonymous or non-synonymous by ANNOVAR. Variants that were present in all cell lines from the same strain background, and variants with a Phred-scaled quality score of < 30 were removed. The exome fastq data have been deposited in NCBI SRA database under accession # SRP096980. A searchable Excel spreadsheet with the processed data is available for download at ftp site ftp://helix.nih.gov/collab/leemax/public/Lalage_Wakefield/paper/exome

### Gene expression analysis

Primary tumors from cells implanted orthotopically into the #4 mammary fatpad of syngeneic mouse hosts were harvested when they reached 0.5-1.0 cm diameter. RNA was prepared from four representative tumors of each of the 12 models using the RNeasy kit (QIAGEN). RNA quality was checked using an Agilent Bioanalyzer and all but two samples (both from 6DT1 model with RIN~7) had a RIN > 8. RNA samples were processed and hybridized to the Affymetrix Mouse Gene 1.0 ST array using standard manufacturer-recommended protocols, and scanned on the Affymetrix GeneChip 3000 Scanner. Raw data were collected using Affymetrix AGCC software. The 48 CEL files were normalized by the RMA method using Affymetrix Expression Console. The probes without gene names were removed and expression values of the multiple probes for the same gene were combined into one by averaging. The unsupervised hierarchical cluster method was applied to divide the 48 tumor samples into three clusters. To derive differentially expressed genes, transcriptomes of tumor samples in one group were compared to those in another group, where each group is one of the clusters or the union of two clusters. The differentially expressed genes from each test (absolute fold-change cutoff > 1.5; *p* < 0.001) were analyzed for pathway enrichment and upstream regulator identification using Ingenuity ^®^ Pathway Analysis (QIAGEN). Upstream regulator analysis in this program is based on prior knowledge of expected effects between transcriptional regulators and their target genes stored in the Ingenuity^®^ Knowledge Base and examines how many known targets of a given transcriptional regulator are present in the dataset as well as comparing the direction of change with that expected from the literature. (http://pages.ingenuity.com/rs/ingenuity/images/0812%20upstream_regulator_analysis_whitepaper.pdf) In an independent analysis, the scores for an EMT signature and an interferon-γ (IFNγ) activation signature were also assessed across the panel. The EMT signature used was a 130 consensus genelist (Supplementary Table 11) derived from a meta-analysis of gene expression signatures defining EMT during cancer progression [53]. The IFNγ signature geneset (Supplementary Table 12) came from a core list of IFNγ-related genes associated with response to immune checkpoint inhibitor therapy (https://www.google.com/patents/WO2015094992A1?cl=en). The EMT score for each sample was defined as the weighted sum of the expression of the core 130 genes, with weights 1 and -1 for up and down indicating the direction of expression change. The IFNγ score for each sample was defined as the sum of the expression of all the genes on the list. Mouse tumor transcriptomes were then compared with the transcriptomes from a published set of human breast cancer patient-derived xenografts [38]. Among the 21201 mouse genes, 15807 have human homologs. The mouse tumor and human PDX gene expression datasets were merged and 13515 genes were found to be shared between the expression datasets. After removing batch effects, the mouse tumor samples and the PDX samples were clustered using the unsupervised hierarchical clustering method. All mouse tumor array data have been deposited in GEO under accession number GSE69006. A searchable Excel spreadsheet containing processed gene expression information data across the panel is also available for download from ftp://helix.nih.gov/collab/leemax/public/Lalage_Wakefield/paper/transcript/.

### Intrinsic subtype analysis

To determine which mouse allograft models map to which intrinsic human subtypes, seven different computational methods were evaluated in combination with four different gene lists to identify the optimal combination for subtype calling across species. The computational methods evaluated were as follows: Genefu, Clustering, K-nearest neighbor (KNN), Support Vector Machine (SVM), Principal component analysis (PCA), General linear models (GLM) and Random forest (RF). The gene lists were the commonly used PAM50 subtyping signature [54], an expanded human “intrinsic” gene list of 1918 genes [54], a mouse-derived intrinsic geneset of 1841 genes [33], and an extended PAM50 gene list (PAM50+) that included the PAM50 geneset plus the 450 genes that overlapped between the mouse and human intrinsic gene lists. To evaluate performance, each of the 28 combinations were trained on the TCGA breast cancer gene expression data and clinical characteristics. Subsequently the trained gene list/computational method combinations were used to call the subtypes of the GSE2034 breast cancer data set, and the results were compared to the pre-existing GSE2034 clinical subtype assignments. The best performing genelist/method combination was then used to assign the mouse tumors to the most closely matched human intrinsic subtype.

## SUPPLEMENTARY MATERIALS FIGURES AND TABLES





## Abbreviations

AUC: area under the curve
CNV: copy number variant
CV: coefficient of variation
DMBA: 7,12-Dimethylbenz[a]anthracene
ER: estrogen receptor
GEMM: genetically engineered mouse model
GEO: Gene Expression Omnibus
GLM: generalized linear model
H&E: hematoxylin and eosin
IPA: Ingenuity Pathway Analysis
KNN: K-nearest neighbor
NBF: neutral buffered formalin
nsSNV: non-synonymous single nucleotide variant
PBS: phosphate-buffered saline
PCA: principle component analysis
PDX: patient-derived xenograft
PR: progesterone receptor
RF: Random Forest
RIN: RNA integrity number
RMA: Robust multi-array average
SNP: single nucleotide polymorphism
sSNV: synonymous single nucleotide variant
SVM: Support Vector Machine
TCGA: The Cancer Genome Atlas
