# Supplementary material for: Immunocompetent mouse allograft models for development of therapies to target breast cancer metastasis
Source: Oncotarget. 2017 Feb 25;8(19):30621–43. doi: 10.18632/oncotarget.15695 (PMC5458155; doi:10.18632/oncotarget.15695)

Immunocompetent mouse allograft models for development of therapies to target breast cancer metastasis

Supplementary Material

Supplementary Figure 1. Histology of primary tumors. Representative H&E stained images of the model panel. Scale bars represent 60μm. Models are ordered alphabetically.

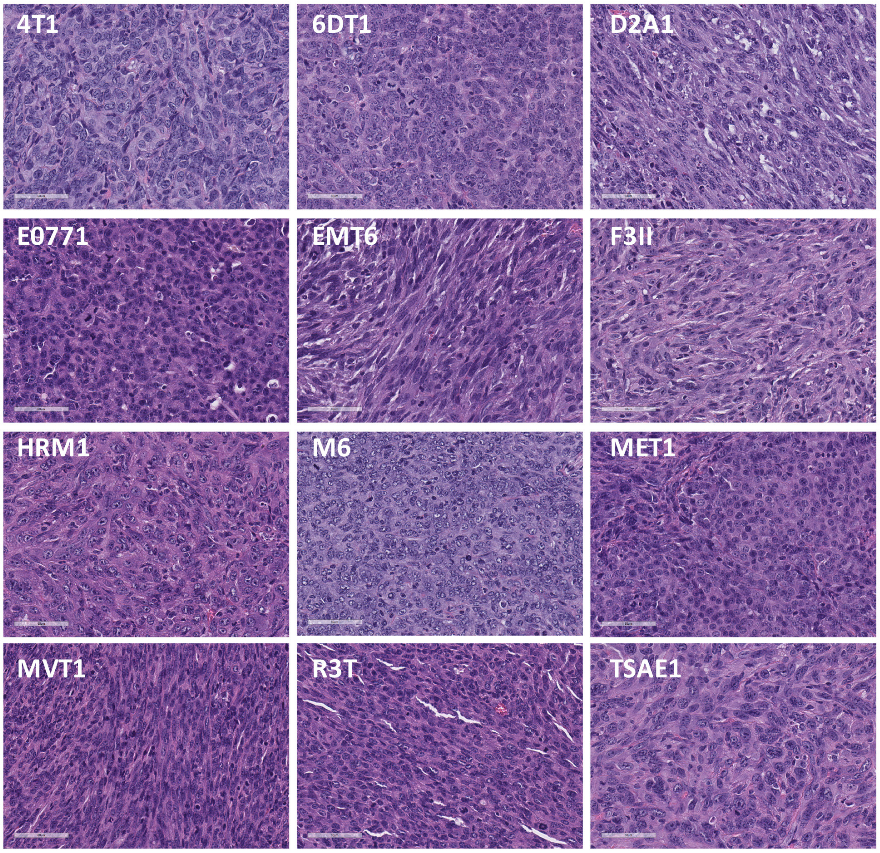

Supplementary Figure 2. Cytokeratin 8 immunostaining of primary tumors. The inset for E0771 shows a normal mammary duct from the same section as an internal positive control. Scale bars represent 200µm. Models are ordered alphabetically.

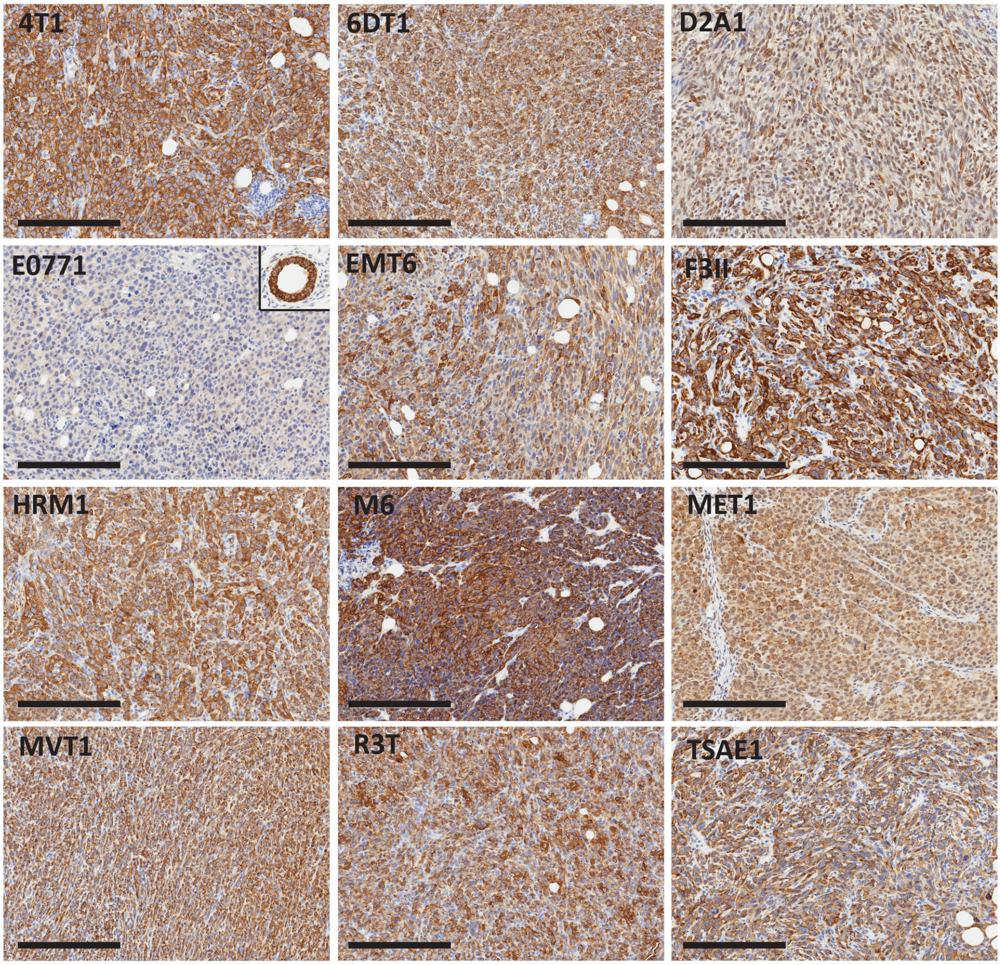

Supplementary Figure 3. Estrogen responsiveness *in vitro* of models that gave rise to estrogen receptor positive tumors *in vivo*. Cells were grown in hormone-depleted medium with or without the addition of 100nM 17-β-estradiol. Culture confluence as a measure of proliferation was assessed by time-lapse imaging as described in Materials and Methods. Only TSAE1 showed significant estrogen responsiveness under these conditions.

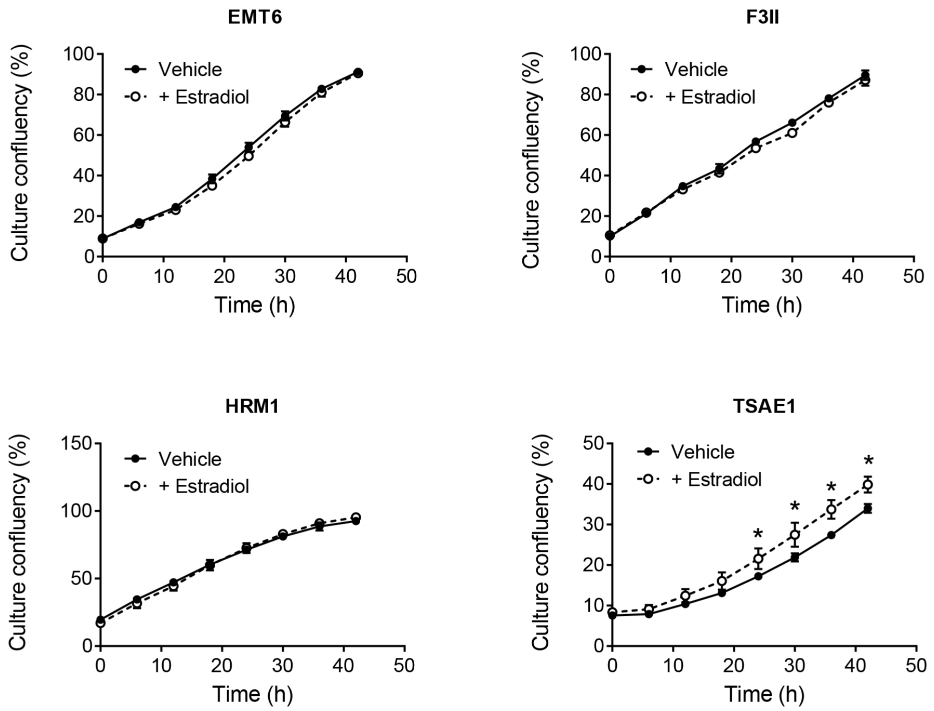

Supplementary Figure 4. Additional immunohistochemical features of the primary tumors. **A.** Aberrant angiogenesis in the MVT1 model. Immunostaining for CD34 in a primary tumor from the MVT1 model (originally derived from an MMTV-Myc/VEGF bitransgenic mouse) shows collapsed vessels with inapparent lumens. The well-vascularized HRM1 primary tumor from a transgenic mouse conditionally expressing a mutant *Pik3ca* is shown for comparison. Scale bars represent 200µm. **B.** Negative correlation between apoptosis and microvessel density in the primary tumors of the model panel (MVT1 model excluded). Each data point represents the mean of 3 individual tumors for a given model. **C.** Trend to higher granulocyte:T-cell ratio (determined by quantitative immunohistochemistry) in primary tumors of cell line models derived from spontaneous (Spont) vs genetically engineered mouse models (GEMM). Meanvalues from 3 tumors for each individual model are plotted. Mann-Whitney test.

A

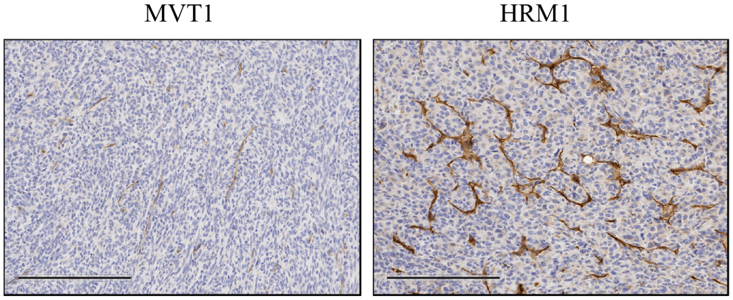

B

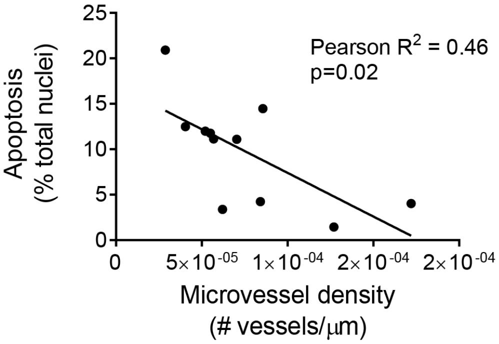

C

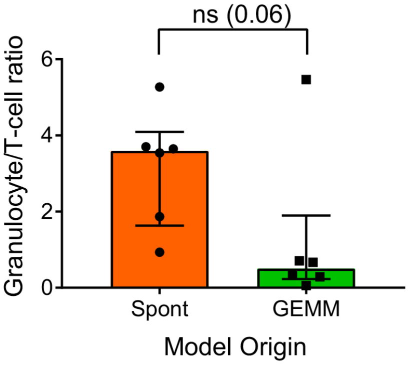

Supplementary Figure 5. Mutation spectrum of nsSNVs for the cell lines of the tumor model panel. The x-axis shows the specific mutation and its 5' nucleotide context. The y-axis shows the incidence of the specific mutation types and their 3'nucleotide contexts. **A.** The E0771 model has a high incidence of A>C transversions that may reflect oxidative stress. **B.** The R3T model shows a preponderance of A>T transversions characteristic of DMBA mutagenesis. **C.** The remaining models have a high incidence of C>T and C>G mutations, as is seen in human breast cancer. Arrows highlight the predominant mutation types.

A E0771 model

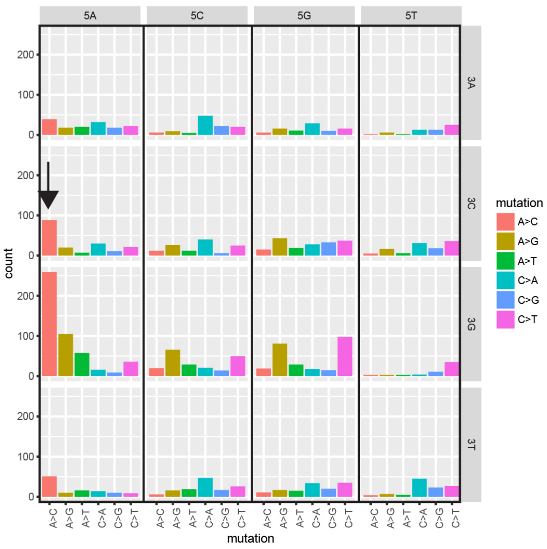

B R3T model

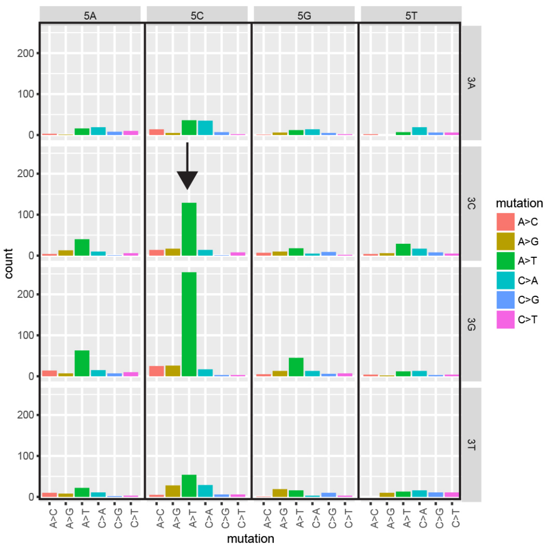

C Entire model panel without R3T and E0771 outliers

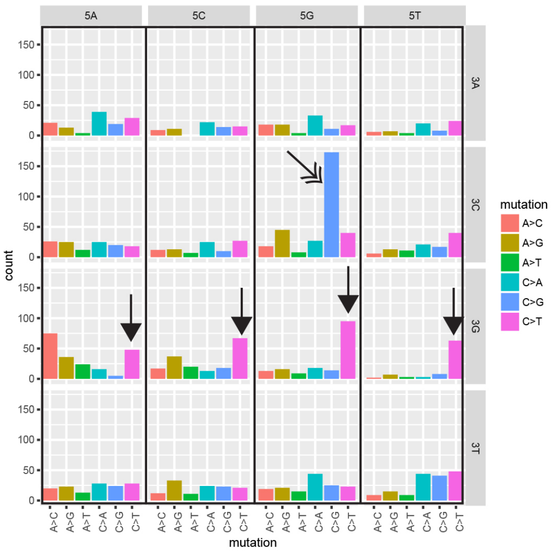

Supplementary Figure 6. p53 functional null status of 4T1 model. Cell cultures *in vitro* were treated with vehicle or adriamycin (0.5μM) for 6h and then Western blots of the cell lysates were probed for p53 and its downstream target p21. MVT1, 6DT1 and M6 are positive controls. p53 is stabilized in untreated M6 cells by the SV40 T-antigen. α-tubulin was the loading control.

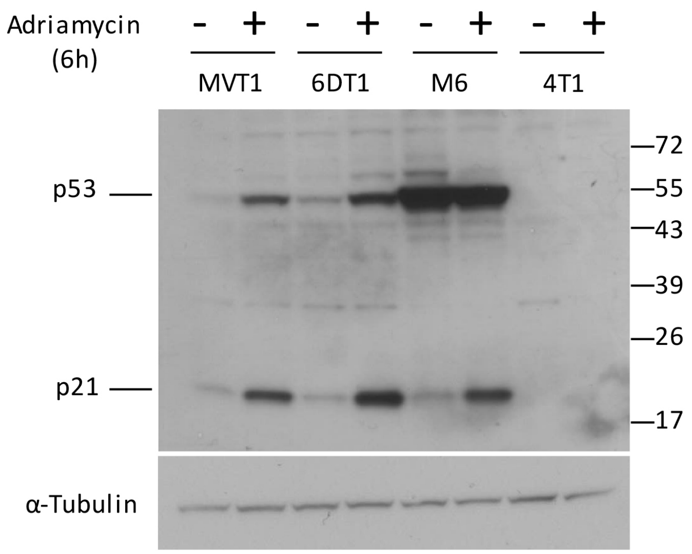

Supplementary Figure 7. Transcriptomic signature scores of individual tumor models and immunohistochemical features of transcriptomic model clusters. A. Interferon-g (IFNg) transcriptomic signature score for the individual primary tumors. Bars are means +/- SD (n=4 individual tumors/model). Models are ordered by signature score and colored by transcriptomic cluster membership or model origin (spontaneous vs genetically engineered mouse model. **B.** Epithelial-to-mesenchymal (EMT) signature score. Conditions as for A. Claudin status of tumors is also indicated. **C.** Leukocyte infiltration of primary tumors as assessed by immunohistochemistry. Each individual data point represents the mean score for 3 tumors/model. Bars show the mean +/- SD for the individual models in each cluster. **D.** Microvessel density in primary tumors assessed by CD34 immunostaining, plotted as for C. One-way ANOVA with Tukey's multiple comparison test. *, $p<0.05$ **E.** Proliferation in primary tumors assessed by Ki67 immunostaining, plotted as for C.

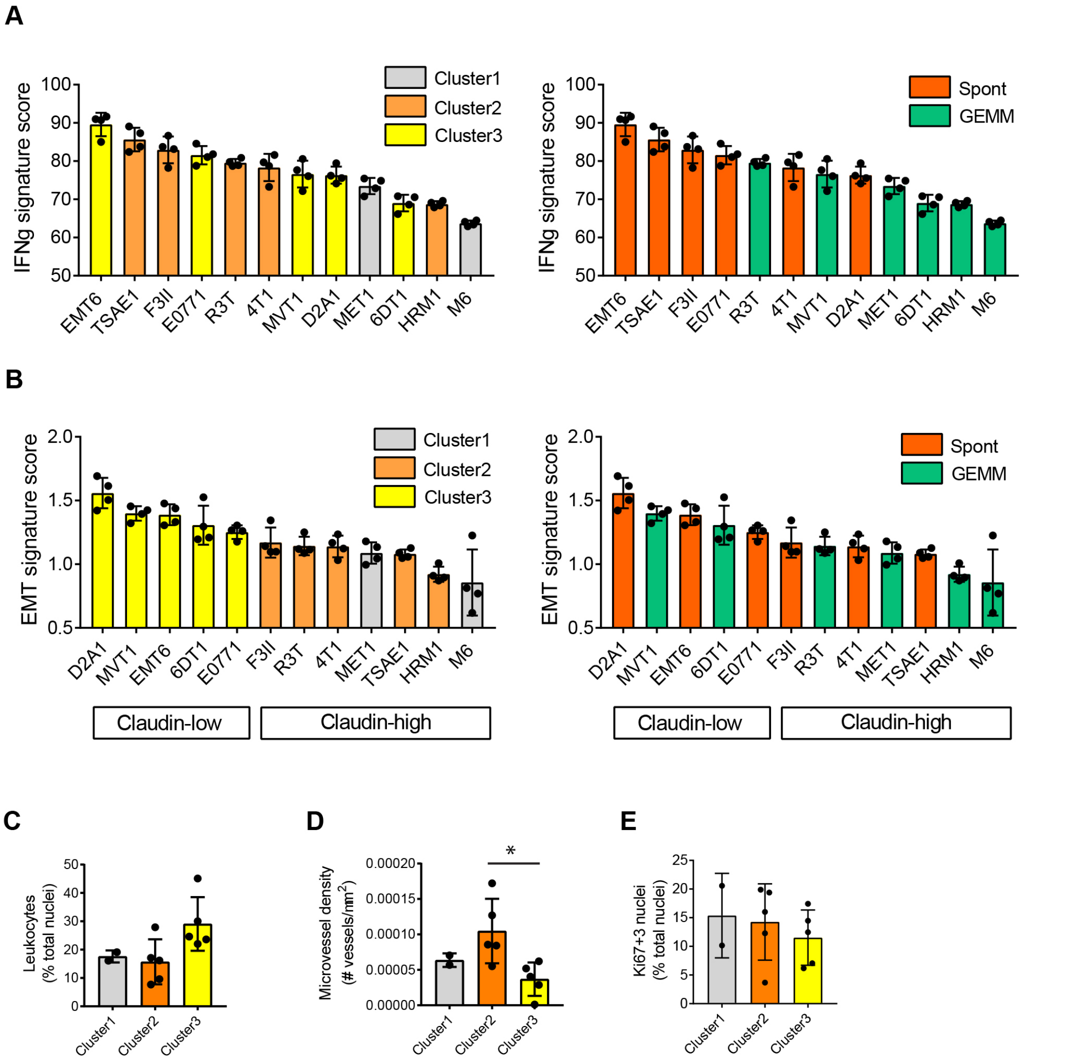

Supplementary Figure 8. Comparison of mean breast cancer subtype calls for orthotopic mouse tumors using either the G1841 or the PAM50 genelists. The intrinsic subtype probability was determined for the tumor model panel using the cluster method with either the G1841 mouse-derived intrinsic gene list (**A)** or the PAM50 genelist (**B**). Models in (A) are ordered by decreasing order of luminal A content. Models in (B) use the same order for direct comparison. The most discrepant models are EMT6 and HRM1.

A

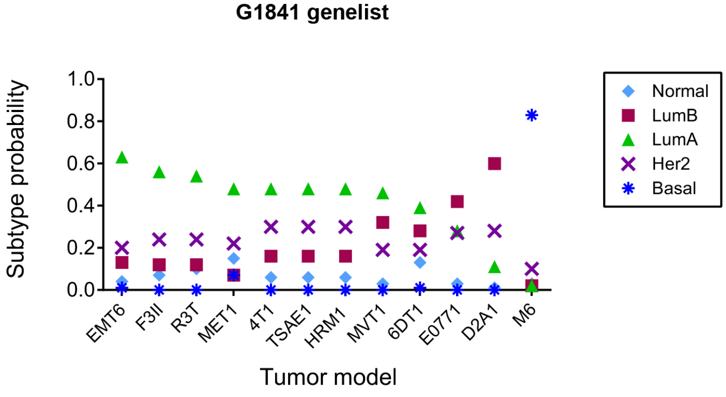

B

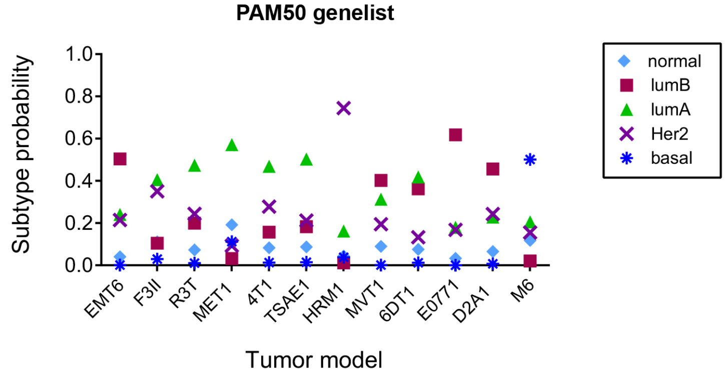

Supplement: Supplementary file 1 [file oncotarget-08-30621-s001.pdf]
