# Supplementary material for: Immunocompetent mouse allograft models for development of therapies to target breast cancer metastasis
Source: Oncotarget. 2017 Feb 25;8(19):30621–43. doi: 10.18632/oncotarget.15695 (PMC5458155; doi:10.18632/oncotarget.15695)
Supplement: Supplementary file 2 [file oncotarget-08-30621-s002.docx]

Immunocompetent mouse allograft models for development of therapies to target breast cancer metastasis

Supplementary Tables

**SUPPLEMENTARY TABLES**

**Supplementary Table 1. Experimental conditions used for metastasis assays in the authors’ lab**

Cell line models that metastasize with <40% efficiency by the orthotopic implantation route are routinely used in the tail vein injection format in the authors’ lab. Note that different substrains of mice from different vendors may give different results, and models should be re-optimized in the facility in which they are to be used. Mfp: mammary fat pad

|  |  | **Experimental conditions for metastasis assay** | | | | | | |
| --- | --- | --- | --- | --- | --- | --- | --- | --- |
| **Cell line** | **Mouse strain** | **Vendor** | **Tumor innoculum (# cells)** | **Route (orthotopic or tailvein)** | **Time to primary tumor resection (days)** | **Time to necropsy (days)** | **Metastatic efficiency (% mice with metastases)** | **Notes** |
| 4T1 | BALB/c | Charles River Labs | 40,000 | Orthotopic | 12-14 | 28 | 100 | #4 mfp |
| 6DT1 | FVB/NCr | Charles River Labs | 50,000 | Orthotopic | 14 | 33 | >90 | #4 mfp |
| D2A1 | BALB/c | Charles River Labs | 250,000 | Orthotopic | 17 | 56 | 45 | #4 mfp; efficiency higher in immunodeficient mice |
| E0771 | C57Bl/6NCr | Charles River Labs | 250,000 | Orthotopic | No resection | 22 | 50 | #2 mfp |
| EMT6 | BALB/c | Charles River Labs | 250,000 | Orthotopic | 13 | 55 | 100 | #4 mfp with Matrigel |
| F311 | BALB/c | Charles River Labs | 500,000 | Tail vein | n/a | 30 | 70 | <40% incidence in orthotopic implantation format |
| HRM1 | FVB/NCr | Charles River Labs | 100,000 | Orthotopic | 14 | 45 | 80 | #2 mfp |
| M6 | FVB/NCr | Charles River Labs | 1,000,000 | Orthotopic | 15 | 55 | 55 | #2 mfp; T-antigen tolerized REAR mice only |
| MET1 | FVB/NCr | Charles River Labs | 200,000 | Tail vein | n/a | 35 | 90 | Highly variable between facilities: in some facilities will only metastasize in immunodeficient mouse hosts |
| MVT1 | FVB/NCr | Charles River Labs | 200,000 | Orthotopic | No resection | 28 | >90 | #4 mfp |
| R3T | 129S1J | Jackson Labs | 200,000 | Orthotopic | No resection | 58 | 60 | #4 mfp |
| TSAE1 | BALB/c | Charles River Labs | 500,000 | Tail vein | n/a | 33 | 100 | <30% incidence in orthotopic implantation format |

**Supplementary Table 2. Histopathology of primary tumors and immunohistochemical marker characteristics**

H&E stained slides for 3 representative primary tumors from each model were read independently by a panel of veterinary pathologists and a human pathologist. Tumors were stained for ER and PR and assigned an Allred score for ER staining. Additional special stains were performed to further characterize tumor cells and distinguish tumor from stromal cells. Scores are for tumor cells. +/- minimal; + mild; ++ moderate; +++ marked; MF, multifocal; D, diffuse; *positive in ribbons/cords of cells but negative elsewhere. †The two tumor models with an Allred score of 2 were designated ER-negative since the staining was of borderline intensity or was very focal, and human tumors showing a similar pattern would be classed as clinically negative.

| **Cell line model** | **Primary tumor diagnosis** | | **Histopathology of primary tumor** | | **Immunohistochemistry score for tumor cells** | | | | | |
| --- | --- | --- | --- | --- | --- | --- | --- | --- | --- | --- |
|  |  |  |  |  | **Hormone receptors** | | | **Special stains** | | |
|  | **Human pathologist** | **Veterinary pathologist** | **Veterinary pathologist description** | **Description from original source publications (where available)** | **ER** | **Allred score** | **PR** | **aSMA** | **CK wide spectrum** | **CK8** |
| 4T1 | Carcinoma, poorly differentiated | Carcinoma, poorly differentiated | Sheets of cells with round or spindle-shaped nuclei, nuclear pleomorphism, karyomegaly, multiple nucleoli and poorly defined amphophilic cytoplasm. Frequent mitoses. Invades fat. Some evidence of poorly differentiated gland formation | Not available | (-) | 0 | (-) | (-) | (+++) D | (+++) D |
| 6DT1 | Carcinoma, poorly differentiated | Carcinoma, poorly differentiated | Sheets of cells with pale nuclei and inapparent or scanty eosinophilic cytoplasm. Tumor cells massively invade fat. In some areas, closely apposed nuclei form ribbons. Scattered mitoses. Morphology is compatible with carcinoma, poorly differentiated but not typical glandular carcinoma | Not available | (-) | 0 | (-) | (+/-) MF | (-) | (+) MF |
| D2A1 | Carcinoma, spindle cell | Carcinoma, sarcomatoid | Whorls and bundles of spindle-shaped cells with eosinophilic cytoplasm and large nuclei with multiple nucleoli. Scattered mitoses. Necrosis, severe, multifocal | Masses of fusiform cells arranged in a loose to tight network and forming sheets, palisading and whorl patterns. | (-) | 0 | (-) | (-) | (+/-) MF | (++) D |
| E0771 | Carcinoma, poorly differentiated | Carcinoma, poorly differentiated | Sheets of roughly cuboidal cells with round nuclei with multiple nucleoli and well-defined eosinophilic cytoplasm. Scattered mitoses. Necrosis, severe, multifocal | Islands of rounded and polyhedral cells, forming occasional poorly shaped glands | (-) | 0 | (-) | (+) MF | (-) | (+/-) MF |
| EMT6 | Carcinoma, spindle cell | Carcinoma, sarcomatoid | Whorls and bundles of spindle-shaped cells with attenuated eosinophilic cytoplasm. Scattered mitoses. Necrosis, severe, multifocal | Saracoma-like pattern with elongated, acidophilic, spindle cells arranged in fascicles and displaying an abundant reticulum. | **(+)** | **3/8** | (-) | (++) D | (+/-) MF | (++) D |
| F311 | Carcinoma, spindle cell | Carcinoma, sarcomatoid | Sheets and whorls of spindle-shaped cells surrounding regions containing fibrin and red blood cells. Nuclei are pleomorphic with multiple nucleoli. Cells have eosinophilic cytoplasm. Numerous mitoses, some atypical. In one tumor, cells were more attenuated resembling sarcoma. One tumor showed evidence of muscle invasion | Invasive sarcomatoid carcinomas mainly composed of spindle cells | **(+)** | **4/8** | (-) | (+++) D | (++) D | (+++) D |
| HRM1 | Carcinoma, poorly differentiated | Carcinoma, poorly differentiated | Sheets of tumor cells invading mucle and fat. Cells have large pleomorphic nuclei with mutiple nucleoli and copious eosinophilic cytoplasm. In many areas, cells form irregular glands or dense clusters. Numerous mitoses, many atypical. | Not available | **(+)** | **3/8** | (-) | (+) MF | (++) MF | (+++) D |
| M6 | Carcinoma, poorly differentiated | Carcinoma, poorly differentiated | Solid tumor with some glandular formation. Tightly apposed basophilic nuclei and inapparent or amphophilic cytoplasm with undefined borders. Areas of spindle-shaped cells suggesting EMT. Necrosis moderate, multifocal | Not available | (-) | 0 | (-) | (-) | (++) MF | (+++) MF |
| MET1 | Carcinoma, poorly differentiated with some comedo-like features | Carcinoma, solid | Primarily solid, but foci of glands scattered throughout. Necrosis, mild to moderate, multifocal | Not available | (-) | 0 | (-) | (-) | (+) MF | (++) MF |
| MVT1 | Carcinoma, spindle cell | Carcinoma, sarcomatoid | Whorls and bundles of spindle-shaped cells with piled up nuclei and inapparent attenuated or eosinophilic cytoplasm. Ribbons of cells and some clusters resembling glands. Scattered mitoses. Areas have sarcomatous morphology. Invasion of fat and/or regional lymph node | Not available | (-) | 2/8† | (-) | (++) MF | (+)* | (++) MF |
| R3T | Carcinoma, spindle cell | Carcinoma, sarcomatoid | Whorls and bundles of cells with spindle-shaped to round nuclei with multiple nucleoli and cytoplasm. Numerous mitoses. | Not available | (-) | 2/8† | (-) | (-) | (+) to (++) MF | (++ to +++) MF |
| TSAE1 | Carcinoma, spindle cell | Carcinoma, sarcomatoid | Sheets and whorls of cells with pleomorphic round or spindle shaped nuclei with multiple nucleoli and eosinophilic cytoplasm with poorly-defined borders. Numerous mitoses, some atypical. Invades fat | Fusiform cells with ovoidal nuclei and large nucleoli. Giant cells, marked angioblastic reaction and collagen production were also noted. | **(+)** | **4/8** | (-) | (+) MF | (++) D | (+++) D |

**Supplementary Table 3. Number of single nucleotide variants in cell lines of the model panel.** Single nucleotide variant (SNV) numbers were determined from exome gDNA sequencing as detailed in Materials and Methods. sSNV, synonymous single nucleotide variant; nsSNV, non-synonymous single nucleotide variant

| **Model** | **Strain** | **Model origin** | **All SNV** | **nsSNV** | **sSNV** | **nsSNV/sSNV** | **Note** |
| --- | --- | --- | --- | --- | --- | --- | --- |
| 4T1 | Balb/c | Spontaneous | 335 | 244 | 91 | 2.68 |  |
| D2A1 | Balb/c | Spontaneous | 553 | 397 | 156 | 2.54 |  |
| EMT6 | Balb/c | Spontaneous | 790 | 565 | 225 | 2.51 |  |
| F311 | Balb/c | Spontaneous | 467 | 327 | 140 | 2.34 |  |
| TSAE1 | Balb/c | Spontaneous | 348 | 265 | 83 | 3.19 |  |
| E0771 | C57Bl/6 | Spontaneous | 4294 | 2944 | 1350 | 2.18 | Tumor originated in 1940 |
| 6DT1 | FVB/N | GEM | 77 | 58 | 19 | 3.05 |  |
| HRM1 | FVB/N | GEM | 160 | 94 | 66 | 1.42 |  |
| M6 | FVB/N | GEM | 299 | 217 | 82 | 2.65 |  |
| MET1 | FVB/N | GEM | 201 | 151 | 50 | 3.02 |  |
| MVT1 | FVB/N | GEM | 87 | 56 | 31 | 1.81 |  |
| R3T | 129S1 | GEM/engineered | 2440 | 1708 | 732 | 2.33 | DMBA mutagenized |
|  |  |  |  |  | **Mean** | **2.49** |  |
|  |  |  |  |  | SD | 0.54 |  |

**Supplementary Table 4. Location and identity of single nucleotide variants in the mouse models for the top 30 genes that are most frequently mutated in human breast cancer.** The list of genes commonly amplified or deleted in human breast cancer was generated from *Lawrence et al, 2014, Nature 505:495-501: Discovery and saturation analysis of cancer genes across 21 tumor types.* Key: 0, absent; 1, heterozygous; 2, homozygous; s SNV, synonymous single nucleotide variant; nsSNV, non-synonymous single nucleotide variant

|  |  |  |  |  |  |  |  |  | **MODEL** | | | | | | | | | | | |
| --- | --- | --- | --- | --- | --- | --- | --- | --- | --- | --- | --- | --- | --- | --- | --- | --- | --- | --- | --- | --- |
| Gene | CHR | POS | REF | ALT | Variant type | Exon | cDNA | Protein | 4T1 | 6DT1 | D2A1 | E0771 | EMT6 | F311 | HRM1 | M6 | MET1 | MVT1 | R3T | TSAE1 |
| Akt1 | chr12 | 112658643 | C | G | ns SNV | 6 | G470C | G157A | 0 | 0 | 0 | 0 | 1 | 0 | 0 | 0 | 0 | 0 | 0 | 0 |
| Arid1a | chr4 | 133685236 | G | T | ns SNV | 18 | C4634A | P1545Q | 0 | 0 | 0 | 0 | 0 | 0 | 1 | 0 | 0 | 0 | 0 | 0 |
| Arid1a | chr4 | 133686435 | G | C | ns SNV | 16 | C3991G | Q1331E | 0 | 0 | 0 | 0 | 1 | 0 | 0 | 0 | 0 | 0 | 0 | 0 |
| Casp8 | chr1 | 58844395 | A | T | ns SNV | 9 | A920T | D307V | 0 | 0 | 0 | 0 | 0 | 0 | 0 | 0 | 0 | 0 | 1 | 0 |
| Cul4b | chrX | 38564101 | T | A | stopgain SNV | 4 | A343T | K115X | 0 | 0 | 0 | 0 | 0 | 0 | 0 | 0 | 1 | 0 | 0 | 0 |
| Erbb2 | chr11 | 98423086 | A | G | ns SNV | 7 | A854G | N285S | 0 | 0 | 0 | 0 | 0 | 0 | 0 | 0 | 1 | 0 | 0 | 0 |
| Kras | chr6 | 145246772 | C | A | ns SNV | 2 | G34T | G12C | 0 | 2 | 0 | 2 | 0 | 0 | 0 | 0 | 0 | 2 | 0 | 0 |
| Kras | chr6 | 145246771 | C | T | ns SNV | 2 | G35A | G12D | 0 | 0 | 0 | 0 | 0 | 0 | 2 | 0 | 0 | 0 | 0 | 2 |
| Map2k4 | chr11 | 65756329 | A | G | ns SNV | 2 | T152C | V51A | 0 | 0 | 0 | 1 | 0 | 0 | 0 | 0 | 0 | 0 | 0 | 0 |
| Med23 | chr10 | 24909827 | T | C | ns SNV | 27 | T3725C | V1242A | 0 | 0 | 0 | 1 | 0 | 0 | 0 | 0 | 0 | 0 | 0 | 0 |
| Ncor1 | chr11 | 62384792 | A | T | ns SNV | 14 | T1563A | D521E | 0 | 0 | 0 | 0 | 0 | 0 | 0 | 0 | 1 | 0 | 0 | 0 |
| Pik3ca | chr3 | 32443673 | C | T | ns SNV | 7 | C1346T | P449L | 0 | 1 | 0 | 0 | 0 | 0 | 0 | 0 | 0 | 1 | 0 | 0 |
| Pten | chr19 | 32811828 | G | T | stopgain SNV | 6 | G625T | G209X | 0 | 0 | 0 | 0 | 2 | 0 | 0 | 0 | 0 | 0 | 0 | 0 |
| Sf3b1 | chr1 | 55007682 | C | G | ns SNV | 7 | G702C | E234D | 0 | 0 | 1 | 0 | 0 | 0 | 0 | 0 | 0 | 0 | 0 | 0 |
| Spen | chr4 | 141475408 | C | T | s SNV | 12 | G5907A | R1969R | 0 | 0 | 1 | 0 | 0 | 0 | 0 | 0 | 0 | 0 | 0 | 0 |
| Spen | chr4 | 141476491 | C | T | s SNV | 12 | G4824A | R1608R | 0 | 0 | 1 | 0 | 0 | 0 | 0 | 0 | 0 | 0 | 0 | 0 |
| Spen | chr4 | 141473335 | C | T | s SNV | 12 | G7980A | P2660P | 0 | 0 | 0 | 0 | 1 | 0 | 0 | 0 | 0 | 0 | 0 | 0 |
| Spen | chr4 | 141471528 | G | A | ns SNV | 12 | C9787T | H3263Y | 0 | 0 | 0 | 1 | 0 | 0 | 0 | 0 | 0 | 0 | 0 | 0 |
| Spen | chr4 | 141477289 | G | A | s SNV | 12 | C4026T | I1342I | 0 | 0 | 0 | 1 | 0 | 0 | 0 | 0 | 0 | 0 | 0 | 0 |
| Tbl1xr1 | chr3 | 22192255 | T | A | s SNV | 9 | T852A | A284A | 0 | 0 | 0 | 2 | 0 | 0 | 0 | 0 | 0 | 0 | 0 | 0 |
| Tbl1xr1 | chr3 | 22189987 | G | A | s SNV | 5 | G424A | A142T | 0 | 0 | 1 | 0 | 0 | 0 | 0 | 0 | 0 | 0 | 0 | 0 |
| Tbx3 | chr5 | 119674049 | T | A | s SNV | 2 | T413A | V138E | 0 | 0 | 0 | 0 | 0 | 0 | 0 | 0 | 1 | 0 | 0 | 0 |
| Trp53 | chr11 | 69588383 | A | G | s SNV | 5 | A386G | K129R | 0 | 0 | 0 | 0 | 0 | 1 | 0 | 0 | 0 | 0 | 0 | 0 |
| Trp53 | chr11 | 69588525 | T | G | ns SNV | 5 | T528G | H176Q | 0 | 0 | 0 | 0 | 0 | 0 | 0 | 0 | 1 | 0 | 0 | 0 |
| Trp53 | chr11 | 69588709 | A | G | ns SNV | 6 | A634G | S212G | 0 | 0 | 0 | 0 | 0 | 0 | 0 | 0 | 0 | 0 | 2 | 0 |
| Trp53 | chr11 | 69589208 | A | T | ns SNV | 7 | A731T | N244I | 0 | 0 | 0 | 0 | 0 | 0 | 0 | 0 | 1 | 0 | 0 | 0 |
| Trp53 | chr11 | 69589629 | G | T | ns SNV | 8 | G830T | R2771 | 0 | 0 | 0 | 0 | 0 | 0 | 0 | 0 | 1 | 0 | 0 | 0 |
| Trp53 | chr11 | 69587267 | G | T | stopgain SNV | 3 | G94T | E32X | 0 | 0 | 0 | 1 | 0 | 0 | 0 | 0 | 0 | 0 | 0 | 0 |
| Trp53 | chr11 | 69589608 | G | A | ns SNV | 8 | G809A | R270H | 0 | 0 | 0 | 0 | 0 | 0 | 0 | 0 | 0 | 0 | 0 | 2 |
| Trp53 | chr11 | 69590672 | G | C | ns SNV | 10 | G1001C | R334P | 0 | 0 | 0 | 1 | 0 | 0 | 0 | 0 | 0 | 0 | 0 | 0 |

**Supplementary Table 5. Incidence in the mouse tumor panel of common copy number variants found in human breast cancer.** The list of genes commonly amplified or deleted in human breast cancer was generated from *Lawrence et al, 2014, Nature 505:495-501. Discovery and saturation analysis of cancer genes across 21 tumor types.* *indicates transgene; ** indicates intronic deletion. Znf217 and Znf703 were not found in the mouse genome. Red indicates amplification and blue indicates deletion.

|  |  | **Mouse model** | | | | | | | | | | | |
| --- | --- | --- | --- | --- | --- | --- | --- | --- | --- | --- | --- | --- | --- |
|  |  | 4T1 | 6DT1 | D2A1 | E0771 | EMT6 | F3II | HRM1 | M6 | MET1 | MVT1 | R3T | TSAE1 |
| Commonly amplified in human BC | Ccnd1 |  |  |  |  |  |  |  |  |  |  |  |  |
|  | Ccnd2 |  |  |  |  |  |  |  |  |  |  |  |  |
|  | Ccne1 |  |  |  |  |  |  |  |  |  |  |  |  |
|  | Egfr |  |  |  |  |  |  |  |  |  |  |  |  |
|  | Erbb2 |  |  |  |  |  |  |  |  |  |  |  |  |
|  | Fgfr1 |  |  |  |  |  |  |  |  |  |  |  |  |
|  | Fgfr2 |  |  |  |  |  |  |  |  |  |  |  |  |
|  | Foxa1 |  |  |  |  |  |  |  |  |  |  |  |  |
|  | Igfr1 |  |  |  |  |  |  |  |  |  |  |  |  |
|  | Mcl1 |  |  |  |  |  |  |  |  |  |  |  |  |
|  | Mdm2 |  |  |  |  |  |  |  |  |  |  |  |  |
|  | Myc |  | * |  |  |  |  |  |  |  | * |  |  |
|  | Ncald |  |  |  |  |  |  |  |  |  |  |  |  |
|  | Notch3 |  |  |  |  |  |  |  |  |  |  |  |  |
|  | Pik3ca |  |  |  |  |  |  |  |  |  |  |  |  |
|  | Tlk2 |  |  |  |  |  |  |  |  |  |  |  |  |
|  | Znf217 |  |  |  |  |  |  |  |  |  |  |  |  |
|  | Znf703 |  |  |  |  |  |  |  |  |  |  |  |  |
| Commonly deleted in human BC | Cdkn2a |  |  |  |  |  |  |  |  |  |  |  |  |
|  | Cdkn2b |  |  |  |  |  |  |  |  |  |  |  |  |
|  | Csmd1 |  |  |  |  |  |  | ** |  |  |  |  |  |
|  | Foxo3 |  |  |  |  |  |  |  |  |  |  |  |  |
|  | Map2k4 |  |  |  |  |  |  |  |  |  |  |  |  |
|  | Mll3 |  |  |  |  |  |  |  |  |  |  |  |  |
|  | Pten |  |  |  |  |  |  |  |  |  |  |  |  |
|  | Ptprd |  |  |  |  |  |  |  |  |  |  |  |  |
|  | Rb1 |  |  |  |  |  |  |  |  |  |  |  |  |
|  | Stk11 |  |  |  |  |  |  |  |  |  |  |  |  |
|  | Trp53 |  |  |  |  |  |  |  |  |  |  |  |  |
|  | Wwox |  |  |  |  |  |  |  |  |  |  |  |  |

**Supplementary Table 6: Evaluation of combination of statistical method and gene set for subtype calling of GSE2034 human breast cancer data set.** Combinations of statistical method and gene set were trained on the TCGA breast cancer dataset and then tested on the GSE2034 breast cancer dataset for their ability to accurately call the intrinsic subtype assignments as described in Methods. PCA= Principal Component Analysis; GLM= Generalized Linear Model; KNN= K-nearest neighbor; RF=Random Forest; SVM= Support Machine Vector; AUC, area under the curve.

| **Method** | **Gene set** | **Accuracy** | **Sensitivity** | **Specificity** | **AUC** |
| --- | --- | --- | --- | --- | --- |
| Cluster | G1841 | 0.910 | 0.902 | 0.565 | 0.930 |
| Cluster | G1918 | 0.915 | 0.908 | 0.568 | 0.924 |
| PCA | G1918 | 0.926 | 0.952 | 0.700 | 0.918 |
| Cluster | PAM50+ | 0.903 | 0.900 | 0.559 | 0.915 |
| PCA | G1841 | 0.919 | 0.927 | 0.708 | 0.912 |
| Genefu | PAM50+ | 0.898 | 0.947 | 0.509 | 0.910 |
| Genefu | G1918 | 0.898 | 0.936 | 0.587 | 0.908 |
| Cluster | PAM50 | 0.893 | 0.903 | 0.562 | 0.899 |
| GLM | G1841 | 0.897 | 0.998 | 0.188 | 0.896 |
| PCA | PAM50+ | 0.915 | 0.933 | 0.615 | 0.894 |
| GLM | PAM50+ | 0.900 | 0.932 | 0.437 | 0.893 |
| GLM | G1918 | 0.889 | 0.997 | 0.173 | 0.887 |
| GLM | PAM50 | 0.900 | 0.928 | 0.512 | 0.881 |
| Genefu | G1841 | 0.904 | 0.948 | 0.545 | 0.879 |
| KNN | G1918 | 0.889 | 0.894 | 0.535 | 0.873 |
| Genefu | PAM50 | 0.892 | 0.945 | 0.515 | 0.872 |
| PCA | PAM50 | 0.903 | 0.931 | 0.596 | 0.871 |
| KNN | G1841 | 0.885 | 0.888 | 0.553 | 0.858 |
| KNN | PAM50 | 0.894 | 0.900 | 0.567 | 0.856 |
| KNN | PAM50+ | 0.894 | 0.899 | 0.567 | 0.855 |
| RF | G1841 | 0.888 | 0.910 | 0.543 | 0.847 |
| RF | PAM50 | 0.887 | 0.902 | 0.547 | 0.843 |
| RF | PAM50+ | 0.890 | 0.906 | 0.548 | 0.837 |
| RF | G1918 | 0.893 | 0.905 | 0.554 | 0.829 |
| SVM | G1841 | 0.866 | 0.909 | 0.496 | 0.819 |
| SVM | G1918 | 0.869 | 0.914 | 0.504 | 0.816 |
| SVM | PAM50 | 0.868 | 0.899 | 0.475 | 0.773 |
| SVM | PAM50+ | 0.858 | 0.904 | 0.410 | 0.738 |

**Supplementary Table 7. Comparison of intrinsic subtype probability calls for the mouse tumor models using different computational methods.** Using the G1841 gene list, the different computational algorithms were used to generate subtype probability calls for the various tumor models. The results are the mean assignment probabilities for 4 primary tumors for each model.

|  | **Clustering** | | | | | **Support Vector Machine** | | | | | **Random Forest** | | | | | **K Nearest Neighbor** | | | | |
| --- | --- | --- | --- | --- | --- | --- | --- | --- | --- | --- | --- | --- | --- | --- | --- | --- | --- | --- | --- | --- |
| **Model** | basal | Her2 | lumA | lumB | normal | basal | Her2 | lumA | lumB | normal | basal | Her2 | lumA | lumB | normal | basal | Her2 | lumA | lumB | normal |
| **4T1** | 0.00 | 0.30 | 0.48 | 0.16 | 0.06 | 0.16 | 0.40 | 0.30 | 0.09 | 0.05 | 0.31 | 0.20 | 0.32 | 0.13 | 0.04 | 0.00 | 0.28 | 0.53 | 0.10 | 0.10 |
| **6DT1** | 0.01 | 0.19 | 0.39 | 0.28 | 0.13 | 0.13 | 0.08 | 0.47 | 0.22 | 0.10 | 0.19 | 0.16 | 0.23 | 0.26 | 0.16 | 0.00 | 0.05 | 0.72 | 0.12 | 0.11 |
| **D2A1** | 0.00 | 0.28 | 0.11 | 0.60 | 0.01 | 0.27 | 0.02 | 0.27 | 0.25 | 0.19 | 0.14 | 0.12 | 0.32 | 0.38 | 0.04 | 0.00 | 0.17 | 0.53 | 0.18 | 0.11 |
| **E0771** | 0.00 | 0.27 | 0.28 | 0.42 | 0.03 | 0.20 | 0.18 | 0.12 | 0.39 | 0.12 | 0.16 | 0.20 | 0.18 | 0.27 | 0.20 | 0.00 | 0.41 | 0.36 | 0.19 | 0.04 |
| **EMT6** | 0.01 | 0.20 | 0.63 | 0.13 | 0.04 | 0.15 | 0.13 | 0.50 | 0.11 | 0.11 | 0.20 | 0.27 | 0.32 | 0.13 | 0.08 | 0.00 | 0.12 | 0.53 | 0.06 | 0.29 |
| **F311** | 0.00 | 0.24 | 0.56 | 0.12 | 0.07 | 0.11 | 0.24 | 0.20 | 0.29 | 0.16 | 0.07 | 0.17 | 0.35 | 0.26 | 0.15 | 0.00 | 0.15 | 0.73 | 0.00 | 0.12 |
| **HRM1** | 0.00 | 0.30 | 0.48 | 0.16 | 0.06 | 0.26 | 0.21 | 0.36 | 0.17 | 0.00 | 0.11 | 0.41 | 0.23 | 0.23 | 0.01 | 0.00 | 0.31 | 0.48 | 0.18 | 0.02 |
| **M6** | 0.83 | 0.10 | 0.02 | 0.02 | 0.03 | 0.25 | 0.10 | 0.62 | 0.03 | 0.00 | 0.26 | 0.24 | 0.17 | 0.25 | 0.07 | 0.18 | 0.22 | 0.40 | 0.08 | 0.13 |
| **Met1** | 0.07 | 0.22 | 0.48 | 0.07 | 0.15 | 0.08 | 0.33 | 0.11 | 0.15 | 0.33 | 0.11 | 0.24 | 0.24 | 0.20 | 0.21 | 0.00 | 0.17 | 0.51 | 0.00 | 0.32 |
| **MVT1** | 0.00 | 0.19 | 0.46 | 0.32 | 0.03 | 0.12 | 0.07 | 0.24 | 0.46 | 0.11 | 0.14 | 0.15 | 0.47 | 0.19 | 0.05 | 0.00 | 0.08 | 0.72 | 0.13 | 0.07 |
| **R3T** | 0.00 | 0.24 | 0.54 | 0.12 | 0.10 | 0.16 | 0.30 | 0.34 | 0.15 | 0.04 | 0.11 | 0.34 | 0.28 | 0.22 | 0.05 | 0.00 | 0.27 | 0.51 | 0.07 | 0.15 |
| **TSAE1** | 0.00 | 0.30 | 0.48 | 0.16 | 0.06 | 0.18 | 0.25 | 0.08 | 0.40 | 0.09 | 0.21 | 0.20 | 0.38 | 0.20 | 0.02 | 0.00 | 0.21 | 0.60 | 0.07 | 0.12 |

|  | **Principal Component Analysis** | | | | | **Genefu** | | | | | **General Linear Model** | | | | |
| --- | --- | --- | --- | --- | --- | --- | --- | --- | --- | --- | --- | --- | --- | --- | --- |
| **Model** | basal | Her2 | lumA | lumB | normal | basal | Her2 | lumA | lumB | normal | basal | Her2 | lumA | lumB | normal |
| **4T1** | 0.10 | 0.44 | 0.20 | 0.13 | 0.13 | 0.32 | 0.22 | 0.21 | 0.03 | 0.23 | 0.00 | 0.26 | 0.27 | 0.22 | 0.25 |
| **6DT1** | 0.11 | 0.34 | 0.25 | 0.14 | 0.16 | 0.17 | 0.06 | 0.51 | 0.14 | 0.12 | 0.00 | 0.22 | 0.30 | 0.22 | 0.26 |
| **D2A1** | 0.11 | 0.49 | 0.15 | 0.13 | 0.12 | 0.48 | 0.07 | 0.12 | 0.12 | 0.21 | 0.00 | 0.28 | 0.25 | 0.26 | 0.22 |
| **E0771** | 0.13 | 0.47 | 0.14 | 0.14 | 0.12 | 0.37 | 0.17 | 0.13 | 0.08 | 0.25 | 0.02 | 0.30 | 0.20 | 0.29 | 0.19 |
| **EMT6** | 0.11 | 0.46 | 0.18 | 0.12 | 0.14 | 0.34 | 0.17 | 0.18 | 0.01 | 0.31 | 0.00 | 0.26 | 0.27 | 0.21 | 0.26 |
| **F311** | 0.11 | 0.37 | 0.23 | 0.13 | 0.15 | 0.06 | 0.34 | 0.42 | 0.03 | 0.15 | 0.00 | 0.25 | 0.28 | 0.22 | 0.25 |
| **HRM1** | 0.10 | 0.47 | 0.18 | 0.13 | 0.12 | 0.47 | 0.26 | 0.00 | 0.13 | 0.14 | 0.00 | 0.29 | 0.25 | 0.26 | 0.20 |
| **M6** | 0.20 | 0.30 | 0.15 | 0.22 | 0.14 | 0.59 | 0.10 | 0.00 | 0.14 | 0.16 | 0.14 | 0.30 | 0.17 | 0.21 | 0.18 |
| **Met1** | 0.12 | 0.29 | 0.27 | 0.17 | 0.16 | 0.10 | 0.38 | 0.20 | 0.22 | 0.10 | 0.00 | 0.25 | 0.29 | 0.21 | 0.26 |
| **MVT1** | 0.09 | 0.49 | 0.19 | 0.12 | 0.11 | 0.01 | 0.09 | 0.64 | 0.06 | 0.21 | 0.00 | 0.25 | 0.27 | 0.25 | 0.23 |
| **R3T** | 0.10 | 0.43 | 0.21 | 0.12 | 0.14 | 0.18 | 0.28 | 0.24 | 0.05 | 0.26 | 0.00 | 0.25 | 0.28 | 0.22 | 0.25 |
| **TSAE1** | 0.10 | 0.44 | 0.20 | 0.13 | 0.13 | 0.28 | 0.25 | 0.21 | 0.03 | 0.22 | 0.00 | 0.28 | 0.26 | 0.24 | 0.23 |

**Supplementary Table 8. Comparison of intrinsic subtype probability calls for the mouse tumor models using different gene lists.** Using the clustering algorithm, the four different genelists were used to generate subtype probability calls for the various tumors models. The results are the mean assignment probabilities for 4 primary tumors for each model.

|  | **G1841 (mouse intrinsic gene list)** | | | | | **G1918 (human intrinsic gene list)** | | | | | **PAM50** | | | | | **PAM50+ (extended PAM50)** | | | | |
| --- | --- | --- | --- | --- | --- | --- | --- | --- | --- | --- | --- | --- | --- | --- | --- | --- | --- | --- | --- | --- |
| **Model** | basal | Her2 | lumA | lumB | normal | basal | Her2 | lumA | lumB | normal | basal | Her2 | lumA | lumB | normal | basal | Her2 | lumA | lumB | normal |
| **4T1** | 0.00 | 0.30 | 0.48 | 0.16 | 0.06 | 0.00 | 0.41 | 0.43 | 0.09 | 0.07 | 0.01 | 0.28 | 0.47 | 0.16 | 0.08 | 0.01 | 0.43 | 0.36 | 0.13 | 0.07 |
| **6DT1** | 0.01 | 0.19 | 0.39 | 0.28 | 0.13 | 0.00 | 0.22 | 0.48 | 0.16 | 0.14 | 0.01 | 0.13 | 0.42 | 0.36 | 0.08 | 0.00 | 0.27 | 0.11 | 0.61 | 0.01 |
| **D2A1** | 0.00 | 0.28 | 0.11 | 0.60 | 0.01 | 0.00 | 0.36 | 0.35 | 0.27 | 0.02 | 0.01 | 0.24 | 0.23 | 0.46 | 0.07 | 0.00 | 0.60 | 0.10 | 0.29 | 0.01 |
| **E0771** | 0.00 | 0.27 | 0.28 | 0.42 | 0.03 | 0.00 | 0.47 | 0.23 | 0.24 | 0.06 | 0.00 | 0.17 | 0.18 | 0.62 | 0.03 | 0.00 | 0.21 | 0.30 | 0.47 | 0.02 |
| **EMT6** | 0.01 | 0.20 | 0.63 | 0.13 | 0.04 | 0.00 | 0.33 | 0.50 | 0.09 | 0.07 | 0.00 | 0.21 | 0.24 | 0.50 | 0.04 | 0.00 | 0.37 | 0.39 | 0.19 | 0.05 |
| **F311** | 0.00 | 0.24 | 0.56 | 0.12 | 0.07 | 0.00 | 0.32 | 0.49 | 0.08 | 0.11 | 0.03 | 0.35 | 0.41 | 0.10 | 0.11 | 0.01 | 0.45 | 0.36 | 0.10 | 0.08 |
| **HRM1** | 0.00 | 0.30 | 0.48 | 0.16 | 0.06 | 0.00 | 0.41 | 0.43 | 0.09 | 0.07 | 0.04 | 0.74 | 0.16 | 0.01 | 0.04 | 0.01 | 0.43 | 0.36 | 0.13 | 0.07 |
| **M6** | 0.83 | 0.10 | 0.02 | 0.02 | 0.03 | 0.38 | 0.27 | 0.20 | 0.03 | 0.12 | 0.50 | 0.16 | 0.21 | 0.02 | 0.12 | 0.67 | 0.22 | 0.04 | 0.01 | 0.06 |
| **Met1** | 0.07 | 0.22 | 0.48 | 0.07 | 0.15 | 0.09 | 0.22 | 0.43 | 0.04 | 0.22 | 0.11 | 0.09 | 0.57 | 0.03 | 0.19 | 0.10 | 0.25 | 0.47 | 0.04 | 0.14 |
| **MVT1** | 0.00 | 0.19 | 0.46 | 0.32 | 0.03 | 0.00 | 0.39 | 0.32 | 0.27 | 0.03 | 0.00 | 0.19 | 0.31 | 0.40 | 0.09 | 0.00 | 0.18 | 0.40 | 0.40 | 0.03 |
| **R3T** | 0.00 | 0.24 | 0.54 | 0.12 | 0.10 | 0.01 | 0.17 | 0.62 | 0.08 | 0.12 | 0.01 | 0.24 | 0.47 | 0.20 | 0.07 | 0.01 | 0.33 | 0.48 | 0.11 | 0.07 |
| **TSAE1** | 0.00 | 0.30 | 0.48 | 0.16 | 0.06 | 0.00 | 0.41 | 0.43 | 0.09 | 0.07 | 0.01 | 0.21 | 0.50 | 0.18 | 0.09 | 0.01 | 0.43 | 0.36 | 0.13 | 0.07 |

**Supplementary Table 9. Growth media and split ratios for cell lines of the tumor panel.** Alpha MEM (Gibco Cat # 11900-024); DMEM, Dulbecco’s Modified Eagle Medium (Gibco Cat # 11995-073); EGF, epidermal growth factor (Gibco Cat # PHG0311); FBS, Fetal bovine serum (Gibco Cat #16000-044); G418 (Gibco Cat # 10131-035); Gentamycin (Gibco Cat # 15710-064); L-glutamine (Gibco Cat # 15030-081); HEPES (Gibco Cat # 15630-080); Insulin (Gibco Cat # 12585-014); Puromycin (Gibco Cat # A11138-03).

| **Cell line** | **Growth Medium** | **Optimal split ratio** |
| --- | --- | --- |
| 4T1 | DMEM, 10% FBS, | 1:8 |
| 6DT1 | DMEM, 10% FBS | 1:5 |
| D2A1 | DMEM, 10% FBS | 1:8 |
| E0771 | RPMI-1640, 5% FBS, 10 mmol/L HEPES | 1:8 |
| EMT6 | DMEM, 10% FBS | 1:6 |
| F311 | DMEM, 5% FBS, 80 mg/L Gentamycin, | 1:8 |
| HRM1 | DMEM/F12, 0.5% FBS, 10 ng/ml EGF | 1:4 |
| M6 | DMEM, 5 % FBS | 1:6 |
| MET1 | DMEM, 10% FBS | 1:2 or 1:3 |
| MVT1 | DMEM, 10% FBS | 1:8 |
| R3T | α-MEM, 8% FBS, 1Xglutamine, 3μg/ml puromycin, 200μg/ml G418 | 1:5 |
| TSAE1 | DMEM, 10% FBS | 1:3 to 1:6 |

**Supplementary Table 10. Immunostaining reagents and conditions used for inmmunohistochemical characterization of the tumor panel.** All immunohistochemistry was performed on formalin-fixed, paraffin-embedded samples. Abbreviations: HIER, heat-induced epitope retrieval: MW AR, microwave antigen retrieval ; AR Citra, Antigen Retrieval Citra (citrate buffer) by BioGenex); PC, pressure cooker; NGS, normal goat serum; 2Ab, secondary antibody; SA, streptavidin; HRP, horseradish peroxidase; M.O.M., Mouse primary antibody on mouse tissue kit (VECTOR); DAB, 3,3'=dioaminobenzidine; ms, mouse; ABC Avidin-Biotinylated Enzyme Complex (VECTOR), RT, room temperature

| **Primary Antibody details** | | | | **Pretreatment** | | **Detection** |  |  |
| --- | --- | --- | --- | --- | --- | --- | --- | --- |
| **Antibody target** | **Primary antibody dilution** | **Manufacturer** | **Catalog #** | **Antigen retrieval** | **Proteolytic digestion** | **Technique/ kit** | **Pos control** | **Chromagen** |
| **Cytokeratin (wide spectrum)** | 1:1000 | DAKO | Z0622 | HIER Citrate 20' | Not used | NGS/2Ab Goat a-rabbit/SA-HRP | Skin | DAB |
| **Cytokeratin 8** | 1:250 | Abcam | ab59400 | MW AR Citra | Not used | NGS/2Ab Goat a-rabbit/ABC | Mammary gland | DAB |
| **Actin, a-Smooth Muscle** | 1:10,000 | Sigma | A2547 | Not used | Not used | UltraVision Quanto Mouse on Mouse HRP Kit | Small intestine | DAB |
| **Vimentin** | 1:500 | Sigma | V6630 | HIER Citrate 20' | Not used | M.O.M. | Kidney | DAB |
| **CD3 (pan T-cell)** | 1:50 | AbD Serotec | MCA1477 | PC AR Citra | Not used | NRS/2Ab Rabbit a-rat ms adsorbed/ABC-AP | Spleen | DAB |
| **F4/80 (clone BM8) (Macrophage)** | 1:100 | eBioscience | 14-4801 (IgG2a) | Not used | Proteinase K 5' RT (DAKO S3020) | NRS/2Ab Rabbit a-rat ms adsorbed/SA-HRP | Spleen | DAB |
| **Ly6G/GR1 (granulocyte)** | 1:50 | Acris | DM3589P | HIER EDTA 10' | Not used | NRS/2Ab Rabbit a-rat ms adsorbed/SA-HRP | Spleen | DAB |
| **CD45 (pan-leukocyte)** | 1:100 | BD Biosciences | 550539 (IgG2b) | Decloak Citra | Not used | NRS/2Ab Rabbit a-rat ms adsorbed/ABC | Spleen | DAB |
| **Estrogen receptor ERa (MC-20)** | 1:500 | Santa Cruz | sc-542 | HIER Citrate 20' | Not used | NGS/2Ab Goat a-rabbit/SA-HRP | Uterus | DAB |
| **CD34 (RAM34) blood vessel** | 1:50 | eBioscience | 14-0341-85 (IgG2a) | HIER EDTA 10' | Not used | NRS/2Ab Rabbit a-Rat ms adsorbed/SA-HRP | Kidney | DAB |
| **Ki-67 (proliferation)** | 1:100 | Abcam | Ab16667 | HIER Citrate 20' | Not used | Bond Polymer Refine Kit | Small intestine | DAB |
| **Caspase-3 (apoptosis)** | 1:250 | Promega | G748A | HIER EDTA 10' | Not used | NGS/2Ab Goat a-rabbit/SA-HRP | Thymus | DAB |
| **Progesterone receptor PR** | 1:200 | DAKO | A0098 | HIER Citrate 20' | Not used | NGS/2Ab Goat a-rabbit/SA-HRP | Uterus | DAB |

**Supplementary Table 11. EMT signature gene list.**

| **Gene** | **Direction** | **Weight** | **Gene** | **Direction** | **Weight** | **Gene** | **Direction** | **Weight** |
| --- | --- | --- | --- | --- | --- | --- | --- | --- |
| ABCA1 | Up | 1 | FN1 | Up | 1 | PRKCA | Up | 1 |
| ABLIM1 | Down | -1 | FST | Down | -1 | PRRG4 | Down | -1 |
| ADAM12 | Up | 1 | FSTL1 | Up | 1 | PRSS8 | Down | -1 |
| ADRB2 | Down | -1 | FXYD3 | Down | -1 | PTGER2 | Up | 1 |
| AGR2 | Down | -1 | GALNT10 | Up | 1 | PTX3 | Up | 1 |
| ALDH1A3 | Down | -1 | GJB3 | Down | -1 | RAPGEF5 | Down | -1 |
| ANK3 | Down | -1 | GPX3 | Down | -1 | RECK | Up | 1 |
| BIK | Down | -1 | HAS2 | Up | 1 | RGS4 | Up | 1 |
| C10orf10 | Down | -1 | IFI30 | Down | -1 | RHOD | Down | -1 |
| C5orf13 | Up | 1 | IGFBP3 | Up | 1 | S100P | Down | -1 |
| CA2 | Down | -1 | IL18 | Down | -1 | SERPINB1 | Down | -1 |
| CD24 | Down | -1 | IL1R1 | Up | 1 | SERPINE1 | Up | 1 |
| CDH1 | Down | -1 | JUP | Down | -1 | SERPINE2 | Up | 1 |
| CDH11 | Up | 1 | KLK10 | Down | -1 | SLC22A4 | Up | 1 |
| CDH2 | Up | 1 | KLK7 | Down | -1 | SLC27A2 | Down | -1 |
| CDK14 | Up | 1 | KRT15 | Down | -1 | SLC7A5 | Down | -1 |
| CDKN2C | Up | 1 | KRT17 | Down | -1 | SLPI | Down | -1 |
| CDS1 | Down | -1 | LAD1 | Down | -1 | SMPDL3B | Down | -1 |
| COL1A1 | Up | 1 | LAMC2 | Up | 1 | SORL1 | Down | -1 |
| COL3A1 | Up | 1 | LOX | Up | 1 | SPINT1 | Down | -1 |
| COL5A1 | Up | 1 | LSR | Down | -1 | SPOCK1 | Up | 1 |
| COL6A1 | Up | 1 | LTBP1 | Up | 1 | SRGN | Up | 1 |
| COL6A3 | Up | 1 | LTBP2 | Up | 1 | ST6GALNAC2 | Down | -1 |
| CTGF | Up | 1 | LUM | Up | 1 | SULF | Up | 1 |
| CTSL2 | Down | -1 | MAP1B | Up | 1 | SYK | Down | -1 |
| CXADR | Down | -1 | MAP7 | Down | -1 | SYNE1 | Up | 1 |
| CXCL16 | Down | -1 | MBP | Down | -1 | SYT11 | Up | 1 |
| CYP1B1 | Up | 1 | MME | Up | 1 | TAGLN | Up | 1 |
| DCN | Up | 1 | MMP2 | Up | 1 | TFPI | Up | 1 |
| DLC1 | Up | 1 | MPZL2 | Down | -1 | TGM2 | Up | 1 |
| DSG3 | Down | -1 | MTUS1 | Down | -1 | TMEM158 | Up | 1 |
| ELF3 | Down | -1 | MYL9 | Up | 1 | TMEM30B | Down | -1 |
| EML1 | Up | 1 | NID2 | Up | 1 | TNFAIP6 | Up | 1 |
| EMP3 | Up | 1 | NR2F1 | Up | 1 | TPD52L1 | Down | -1 |
| EPCAM | Down | -1 | NRP1 | Up | 1 | TPM1 | Up | 1 |
| EPHA1 | Down | -1 | OCLN | Down | -1 | TSPAN1 | Down | -1 |
| FAM169A | Down | -1 | OVOL2 | Down | -1 | TUBA1A | Up | 1 |
| FBLN1 | Up | 1 | PKP2 | Down | -1 | VCAN | Up | 1 |
| FBLN5 | Up | 1 | PLAT | Up | 1 | VIM | Up | 1 |
| FBN1 | Up | 1 | PLS1 | Down | -1 | WNT5A | Up | 1 |
| FGF2 | Up | 1 | PLXNB1 | Down | -1 | ZEB1 | Up | 1 |
| FGFR1 | Up | 1 | PMP22 | Up | 1 | ZHX2 | Down | -1 |
| FGFR2 | Down | -1 | PPAP2B | Up | 1 | ZNF165 | Down | -1 |
| FGFR3 | Down | -1 | PPL | Down | -1 |  |  |  |

**Supplementary Table 12. Interferon-γ genelist**

| **Gene** | **Direction** | **Weight** |
| --- | --- | --- |
| IFNG | Up | 1 |
| STAT1 | Up | 1 |
| CCR5 | Up | 1 |
| CXCL9 | Up | 1 |
| PRF1 | Up | 1 |
| HLA-DRA | Up | 1 |
| CXCL10 | Up | 1 |
| CXCL11 | Up | 1 |
| IDO1 | Up | 1 |
| GZMA | Up | 1 |
